# Porcine mesothelium matrix as a biomaterial for wound healing applications

**DOI:** 10.1016/j.mtbio.2020.100057

**Published:** 2020-05-17

**Authors:** H. Capella-Monsonís, M.A. Tilbury, J.G. Wall, D.I. Zeugolis

**Affiliations:** a Regenerative, Modular & Developmental Engineering Laboratory (REMODEL), National University of Ireland Galway (NUI Galway), Galway, Ireland; b Science Foundation Ireland (SFI) Centre for Research in Medical Devices (CÚRAM), National University of Ireland Galway (NUI Galway), Galway, Ireland; c Department of Microbiology, National University of Ireland Galway (NUI Galway), Galway, Ireland

**Keywords:** Xenografts, Collagen devices, Functional biomaterials, Immune response, Angiogenesis, CORC-PG, collagen/oxidized regenerated cellulose—Promogran™, DMEM, Dulbecco's modified eagle medium, ECM, extracellular matrix, HUVECs, human umbilical vein endothelial cells, LB, lysogenic broth, LPS, lipopolysaccharides, OF-EF, ovine forestomach—Endoform™, P/S, penicillin/streptomycin, PBS, phosphate-buffered saline, PFA, paraformaldehyde, PM-MB, porcine mesothelium—Meso Biomatrix®, PM-PC, porcine mesothelium—Puracol® Ultra ECM, PUB-MS, porcine urinary bladder—MatriStem®, SDS-PAGE, sodium dodecyl sulphate–polyacrylamide gel electrophoresis

## Abstract

The increasing economic burden of wound healing in healthcare systems requires the development of functional therapies. Xenografts with preserved extracellular matrix (ECM) structure and biofunctional components overcome major limitations of autografts and allografts (e.g. availability) and artificial biomaterials (e.g. foreign body response). Although porcine mesothelium is extensively used in clinical practice, it is under-investigated for wound healing applications. Herein, we compared the biochemical and biological properties of the only two commercially available porcine mesothelium grafts (Meso Biomatrix® and Puracol® Ultra ECM) to traditionally used wound healing grafts (Endoform™, ovine forestomach and MatriStem®, porcine urinary bladder) and biomaterials (Promogran™, collagen/oxidized regenerated cellulose). The Endoform™ and the Puracol® Ultra ECM showed the highest (*p*<0.05) soluble collagen and elastin content. The MatriStem® had the highest (*p*<0.05) basic fibroblast growth factor (FGFb) content, whereas the Meso Biomatrix® had the highest (*p*<0.05) transforming growth factor beta-1 (TGF-*β*1) and vascular endothelial growth factor (VEGF) content. All materials showed tissue-specific structure and composition. The Endoform™ and the Meso Biomatrix® had some nuclei residual matter. All tissue grafts showed similar (*p*>0.05) response to enzymatic degradation, whereas the Promogran™ was not completely degraded by matrix metalloproteinase (MMP)-8 and was completely degraded by elastase. The Promogran™ showed the highest (*p*<0.05) permeability to bacterial infiltration. The Promogran™ showed by far the lowest dermal fibroblast and THP-1 attachment and growth. All tested materials showed significantly lower (*p*<0.05) tumor necrosis factor-alpha (TNF-α) expression than the lipopolysaccharides group. The MatriStem® and the Puracol® Ultra ECM promoted the highest (*p*<0.05) number of micro-vessel formation, whereas the Promogran™ the lowest (*p*<0.05). Collectively, these data confer that porcine mesothelium has the potential to be used as a wound healing material, considering its composition, resistance to enzymatic degradation, cytocompatibility, and angiogenic potential.

## Graphical abstract

**Figure:**
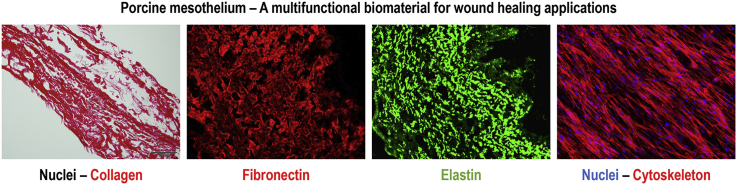

## 1 Introduction

Wound healing represents a substantial financial burden in current healthcare systems with estimated annual healthcare expenditure in excess of $50 billion in the United States alone [1]. The estimated global prevalence is over 3.5 per 100,000 people, which continuously raises, as life expectancy and disease associated non-healing conditions (e.g. diabetes) increase [2,3]. It is thus urgent and imperative to develop functional therapies for wound healing applications.

Decellularized xenografts have shown promise in wound healing management [[4], [5], [6], [7], [8]], overcoming disadvantages of human grafts (e.g. low availability, donor site morbidity) and synthetic biomaterials (e.g. foreign body response). Yet again, there is no consensus on the ideal xenograft, considering the scattered therapeutic efficacy and efficiency (e.g. the porcine dermal matrix Permacol™ in hernia [9,10] repair, the porcine small intestine submucosa CorMatrix® in pediatric cardiovascular surgery [11,12] and the porcine dermal matrix Strattice® in breast reconstruction [13,14] have shown both positive and negative results).

Porcine mesothelium is a tissue rich in connective tissue (CT, e.g. collagens types I and III, elastin, fibronectin) and basement membrane (BM, e.g. collagen type IV and laminin) proteins and growth factors (e.g. FGF-2, TGF-*β*, VEGF) [[15], [16], [17]]. These extracellular matrix (ECM) components present recognition motifs that promote the attachment and proliferation of cells [[18], [19], [20]], contributing to the high cytocompatibility and low immunogenicity *in vitro* of porcine mesothelium [21,22] and allowing re-epithelialization *in vitro*, promoted by its BM components [22,23]. Furthermore, growth factors retained within the porcine mesothelium matrix promote wound healing events, such as cell proliferation and angiogenesis *in vivo* [22,24,25]. Such features clearly illustrate their potential in the wound healing scenario, where cell proliferation, re-epithelialization, and angiogenesis are desirable events to be promoted. Despite all these advantages, commercially available porcine mesothelium grafts have only been used in breast [13], cartilage [26], and nasal [27] reconstruction and as a tendon protector sheet [21].

Herein, we compared the biochemical and biological properties of the only two commercially available porcine mesothelium grafts [Meso Biomatrix® and Puracol® Ultra ECM (PM-MB and PM-PC)] to traditionally used wound healing grafts [ovine forestomach—Endoform™ (OF-EF) [28] and porcine urinary bladder—MatriStem® (PUB-MS) [29,30]] and biomaterials [collagen/oxidized regenerated cellulose—Promogran™ (CORC-PG) [[31], [32], [33], [34]]] that have also shown efficiency and efficacy in wound healing clinical trials.

## 2 Materials and methods

The products assessed in this study are provided in Table 1. All chemicals and consumables were purchased from Sigma-Aldrich (Ireland), unless otherwise stated.

**Table 1:** Commercially available products that were assessed in this study.

Product description & name
Collagen/Oxidized regenerated cellulose—Promogran™ (CORC-PG), Acelity™, USA
Ovine forestomach—Endoform™ (OF-EF), Hollister Wound Care, USA
Porcine urinary bladder—MatriStem® (PUB-MS), ACell®, USA
Porcine mesothelium—Meso Biomatrix® (PM-MB), DSM Biomedical, Netherlands
Porcine mesothelium—Puracol® Ultra ECM (PM-PC), Medline Industries, USA

### 2.1 Sodium dodecyl sulphate–polyacrylamide gel electrophoresis

The presence of soluble collagen type I was assessed with sodium dodecyl sulphate–polyacrylamide gel electrophoresis (SDS-PAGE) [35]. Briefly, small pieces of each material were cut, weighed, and incubated in 1 mg/mL pepsin (P6687, Sigma-Aldrich, Ireland) in 0.5M acetic acid overnight at 4 °C under continuous agitation (1 mg of material per 1 mL of pepsin/acetic acid solution). Solutions were then centrifuged (Heraeus Fresco 17 Centrifuge, Thermo Fisher, Ireland) at 13,000 rpm and 4 °C for 15 min, supernatants were recovered and loaded onto a Mini-Protean 3 SDS-PAGE unit (Bio-Rad Laboratories, UK). Three percent stacking and 5% separation gels were used. Purified collagen type I (CBP2US, Symatese, France) was used as standard. Gels were stained using the SilverQuest™ Silver Staining kit, as per manufacturer's protocol (Thermo Fisher, Ireland).

### 2.2 Elastin and collagen quantification

Elastin content was quantified using the Fastin™ Elastin Kit (Biocolor, UK), as per manufacturer's protocol. The total amount of collagen in each material was analyzed by hydroxyproline assay [35]. Briefly, 5 mg of each sample were hydrolyzed in 6M HCl at 110 °C overnight. The hydrolysates were then centrifuged (Heraeus Pico 17 Centrifuge, Thermo Fisher, Ireland) at 15,000 g and room temperature for 10 min and 10×, 50×, and 100× dilutions of the supernatants were prepared. One hundred ten microliters of these dilutions were transferred to a microcentrifuge tube and 176 μl of chloramine-T reagent were added. The samples were then mixed and incubated for 10 min at room temperature. After incubation, 460 *μ*l of Ehrlich's reagent were added, the samples were vortexed (Fisherbrand™ Classic Vortex Mixer, Thermo Fisher, Ireland) for 30 s and incubated at 70 °C for 10 min. Then, 200 *μ*l of each sample were transferred to a well of a 96-well plate and absorbance (Varioskan Flash Spectral Scanning Multimode Reader, Thermo Fisher, Ireland) was measured at 555 nm. The hydroxyproline corresponding to the elastin (1% wt/wt) was subtracted from the total hydroxyproline content. The remaining hydroxyproline amount was used to calculate the collagen content by dividing by 0.135 (13.5% wt/wt) [35].

### 2.3 Growth factor quantification

The content of growth factors was assessed using ELISA [22]. Briefly, samples were weighed and proteins were extracted using a radioimmunoprecipitation assay extraction buffer (R0278, Sigma-Aldrich, Ireland) with a protease inhibitor cocktail (P9599, Sigma-Aldrich, Ireland). To each sample, 1 mL of extraction buffer was added and samples were incubated in a tissue homogenizer (TissueLyser LT, Qiagen, UK) overnight at 50 rpm and 4 °C. Samples were then centrifuged (Heraeus Fresco 17 Centrifuge, Thermo Fisher, Ireland) at 13,000 rpm and 4 °C for 15 min. Supernatants were then concentrated using Pierce™ 3K Concentrators (Thermo Fisher, Ireland), and basic fibroblast growth factor (FGF-basic), vascular endothelial growth factor (VEGF), and transforming growth factor beta-1 (TGF-*β*1) content was measured using ELISA DuoSet® kits (DY233, DY293B and DY240, respectively; R&D Systems, UK), as per manufacturer's protocols.

### 2.4 Histology and immunohistochemistry analysis

For further compositional analysis, samples were cut into 1 cm^2^ pieces, hydrated for 2 h in phosphate-buffered saline (PBS) at room temperature and then stored at −80 °C in Tissue Freezing Medium® (Leica Biosystems, Ireland). Transverse cryosections of 5 μm thickness were obtained using a CM1850 Cryostat (Leica Biosystems, Ireland) operating at −20 °C. The cryo-sections were subsequently stained with hematoxylin/eosin, Picrosirius red and Masson's trichrome using DPX mountant (06522, Sigma-Aldrich, Ireland) [36].

Immunohistochemistry analysis was carried out for collagen type I (ab90395, Abcam, USA), collagen type III (ab7778, Abcam, USA), collagen type IV (ab6586, Abcam, USA), elastin (ab21610, Abcam, USA), laminin (L939, Sigma-Aldrich, Ireland), and fibronectin (F7387, Sigma-Aldrich, Ireland) [36]. Cryosections were blocked at room temperature with 5% normal goat serum and 0.1% Triton X-100 in PBS for 1 h. The sections were then incubated with the primary antibodies diluted in blocking buffer overnight at 4 °C, followed by three washes in PBS at room temperature. Subsequently, secondary antibodies at 1:500 in blocking buffer were added (Alexa Fluor 488 goat anti rabbit and Alexa Fluor 555 goat anti mouse, Life Technologies, Ireland) for 1 h at room temperature, followed by three washes in PBS at room temperature. To assess whether any cellular remnants had remained, sections were stained with Hoechst (H1399, Invitrogen, Ireland) at 1:5,000 in PBS for 5 min at room temperature. Sections were then mounted with Fluoromount™ Aqueous Mounting Medium (F4680, Sigma-Aldrich, Ireland), left for 2 h at room temperature and then stored at 4 °C. Images were taken with an inverted fluorescence microscope (IX81, Olympus, UK).

### 2.5 Enzymatic degradation

Resistance to collagenase [35] and elastase [37] degradation was also assessed. Briefly, 5 mg pieces of each material were cut and placed into Eppendorf tubes. One milliliter of Tris–HCl buffer pH 7.40 containing 50 U/mL of matrix metalloproteinase (MMP)-8 (17101015, Gibco®, Ireland) or Tris buffer pH 8.5 containing 0.1 U/mL of elastase (E7885, Sigma-Aldrich, Ireland) was added. The samples were then incubated at 37 °C under agitation in an orbital shaker (MaxQ 4000, Thermo Fisher, Ireland) at 150 rpm for 2, 4, 8, 12, and 24 h. The solubilized portion was discarded after centrifugation (Heraeus Pico 17 Centrifuge, Thermo Fisher, Ireland) at 13,000 rpm and room temperature for 10 min and the remaining pellets where weighed after overnight freeze drying (FreeZone Plus 4.5, Labconco, Thermo Fisher, Ireland). The percentage of weight loss over time was subsequently calculated for each material and enzyme.

### 2.6 Swelling ratio analysis

Pieces from all the materials were cut with an 8-mm-diameter biopsy punch and were weighed with a laboratory scale (MH-124, Fisherbrand, UK). The materials were then incubated in PBS overnight at room temperature. After blotting excess PBS with Whatman filter paper, their weight was recorded. Swelling (%) was calculated as (wet weight – dry weigh)/dry weight %.

### 2.7 Bacterial penetration assay

Microbial analysis was conducted using *Escherichia coli* [*E. coli*, BL21(DE3), Invitrogen, Ireland] [38,39]. To assess the effect of the different materials on bacterial growth, bacteria were seeded on lysogenic broth (LB) agar petri dishes at 10^10^ CFU/mL and allowed to dry for 10 min at room temperature. Then, 6 mm discs of each material were soaked in sterile PBS for 20 min, placed on the agar plates, incubated at 37 °C for 24 h and the inhibition growth area was measured using ImageJ (NIH, USA). Filter paper discs loaded with 50 μg of ampicillin sodium salt (A9518, Sigma-Aldrich, Ireland) were used as control. To assess the penetration of bacteria in the materials, trans-well constructs attached to a silicone sheet were used. The silicone sheet between the inner and outer layers was perforated with a 6 mm biopsy punch, and 13 mm discs of each materials were fixed on the silicone sheet using glue. The hole was covered and the materials formed the only barrier between the chambers; care was taken so the glue was not deposited in the hole/material area. The constructs were sterilized under UV for 1 h and 70% ethanol for 30 min, followed by three washes of PBS. A single colony of *E. coli* from an agar plate was used to inoculate 50 mL LB and grown with continuous agitation at 37 °C until the culture reached an optical density (OD)_600_ of 0.7–0.8. The culture was centrifuged (Heraeus Pico 17 Centrifuge, Thermo Fisher, Ireland) at 6,000 rpm for 5 min at room temperature, the pellet was resuspended in sterile PBS, and 0.5 mL of the suspension containing 10^10^ CFU/mL *E. coli* was added to the inner chamber of the trans-well. In the outer chamber, 1 mL of sterile PBS was placed, and aliquots of 50 *μ*l were taken after incubation for 1, 2, and 4 h at 37 °C with mild agitation. Aliquots were then serially diluted and plated on LB agar plates at 10^−1^, 10^−5^, and 10^−8^ dilutions and the number of CFU were counted after incubation at 37 °C for 24 h. In a pilot study, it was confirmed that the industrial glue and system used did not affect the viability of the bacteria or the ability of the unperforated silicone sheet without perforation to contain the microorganisms. After 24 h incubation, the materials were fixed in 4% paraformaldehyde (PFA, 158127, Sigma-Aldrich, Ireland) and cryosections were prepared as described in Section 2.4. Cryosections were stained with 4′,6-diamidino-2-phenylindole (DAPI) to qualitatively assess the localization of the bacteria within the material.

### 2.8 Dermal fibroblast response analysis

Cytocompatibility was assessed using primary adult dermal fibroblasts (PCS-201-012, ATCC®, UK). CORC-PG, OF-EF, PUB-MS, PM-MB, and PM-PC were cut into 1 cm^2^ pieces, placed at the bottom of 24-wellplates and fixed with a silicone O-ring (Z504165, Sigma-Aldrich, Ireland). Then, they were sterilized with 70% ethanol for 30 min at room temperature and washed three times with PBS. Dulbecco's modified eagle medium (DMEM) supplemented with 10% of foetal bovine serum (FBS) and 1% penicillin/streptomycin (P/S) containing 15,000 cells/mL was gently poured on top of the CORC-PG, both sides of the OF-EF [serosa (SR) and papillae (PL)] and both sides of the PUB-MS, PM-MB, and PM-PC (CT and BM), and incubated at 37 °C and 5% CO_2_ for 3, 7, and 14 days. Media were changed every three days. Cell morphology was evaluated after fixation with 4% PFA for 15 min at room temperature and rhodamine/phalloidin (R415, Life Technologies, Ireland) and Hoechst (62249, Thermo Fisher, Ireland) staining. Images were taken with an inverted fluorescence microscope (IX81, Olympus, UK). Nuclei counting was used to assess cell proliferation. Cell metabolic activity and viability were evaluated at each time point with alamarBlue® (Thermo Fisher, Ireland) and LIVE/DEAD® (Thermo Fisher, Ireland) assays, respectively. Metabolic activity was first normalized to cell number and then expressed relatively to the control tissue culture plate (TCP). Cell proliferation was expressed relatively to the control TCP.

### 2.9 Monocyte response analysis

Immune response was assessed using monocyte-like cells (THP-1, TIB-202, ATCC®, UK) [21]. Briefly, cells were expanded in suspension in RPMI-1640 medium with 10% FBS and 1% PS (Sigma-Aldrich, Ireland). Then, THP-1 cells were seeded on the materials at 25,000 cells/cm^2^. To induce macrophage phenotype, cells were treated with phorbol 12-myristate 13-acetate (P8139, Sigma-Aldrich, Ireland) at 100 ng/mL for 24 h at 37 °C and 5% CO_2_. Non-attached cells were washed with PBS and seeded cells were incubated with complete RPMI-1640 medium. As positive inflammatory control, cells were treated with lipopolysaccharides (LPS) from *E. Coli* (L2637, Sigma-Aldrich, Ireland) at 100 ng/mL. All conditions were in culture for 1 and 2 days. Cell metabolic activity, viability, proliferation, and morphology of cells were assessed, as described in Section 2.7. Released tumor necrosis factor-alpha (TNF-*α)* in the medium was quantified using an ELISA assay (DY210, R&D Systems, UK). Same experiments were also performed on THP-1 attached to TCP and then treated with conditioned media, which were prepared by incubating media with each material for 48 h at 37 °C under continuous agitation, and subsequent filtering with a 0.2 μm Millipore filter.

### 2.10 Scratch assay

The *in vitro* angiogenic potential of all materials was assessed using the scratch assay [40]. Human umbilical vein endothelial cells (HUVECs, C2517A, Lonza, UK) were expanded in specific medium (EGM™-2, Lonza, Ireland). When they reached 85–90% confluence, they were seeded in 48-wellplates and incubated at 37 °C and 5% CO_2_ until confluent (2 days). Using a sterile pipette tip, 1 mm wide gap was created at the cell monolayer. The cells were then washed three times with PBS to remove cellular debris and treated with medium conditioned with each material. Conditioned media was created by incubating supplemented with 2% FBS and 1% P/S DMEM with each of the materials at 20 mg/mL overnight at 37 °C under continuous agitation in an incubated orbital shaker (MaxQ 4000, Thermo Fisher, Ireland) at 150 rpm. These mixtures were then sterile filtered and poured on the cell monolayer with the gap. DMEM with 2% FBS and 1% P/S and endothelial growth medium (EGM™-2, Lonza, Ireland) were used as negative and positive controls, respectively. Images were taken at 4, 8, 12, and 24 h and the area fold change in the monolayer moving into the scratch zone with respect to the area at time 0 was calculated for each material.

### 2.11 Rat aortic ring assay

The aortic ring assay was carried out to compare the impact on angiogenesis of the different materials in an *ex vivo* model [40]. The preclinical work was conducted as per NUI Galway's rules and regulations governing preclinical assessment, following the internationally established 3Rs principles. Animals were used from the study with approval number 17Apr01 (Animal Care Research Ethics Committee, NUI Galway). Briefly, three adult (12 weeks) female Sprague Dawley rats were housed with water and food *ad libitum*. The rats were euthanized by isoflurane overdose and decapitation. The aortas were dissected, cleaned, and sectioned into 2-mm-thick sections. Remaining biological waste was frozen, sterilized, and disposed according to NUI Galway’s biological waste management policies. Five hundred microliters of the different conditioned media (see Section 2.9), containing fibrinogen (F4883, Sigma-Aldrich, Ireland) at 3 mg/mL, were used to cover the aortic rings in a 24-well plate. Then, thrombin (T1063, Sigma-Aldrich, Ireland) at 1 U/mL was added to form a hydrogel. Gels were set overnight at 37 °C and 5% CO_2_ and then 500 μL of the correspondent conditioned media was gently poured over the gels. DMEM supplemented with 2% FBS and 1% P/S was used as negative control, whereas DMEM supplemented with 100 ng/mL of VEGF (AF-100-20, PeproTech, UK) was used as positive control. The aorta rings in the gels were then incubated at 37 °C and 5% CO_2_, and images were taken at 4× magnification after 3 and 5 days using an inverted microscope (EVOS® Image System, Thermo Fisher, Ireland). ImageJ (NIH, USA) was used to measure the megapixels of the new micro-vessels formed by creating masks.

### 2.12 Statistical analysis

Data were analyzed using the IBM SPSS Statistics software (IBM Analytics, USA). Student's *t*-test and one-way analysis of variance followed by Fisher's post-hoc test were used after confirming normal distribution of the populations (Kolmogorov–Smirnov) and the equality of variances (Levine's test for homogeneity of variance). For non-normal distributions or different variances, Mann–Whitney *U* test and Kruskal–Wallis test were used to assess significant differences. Statistical significance was accepted at *p*<0.05.

## 3 Results

### 3.1 SDS-PAGE, content of collagen, elastin, and growth factors

**Fig. 1:**
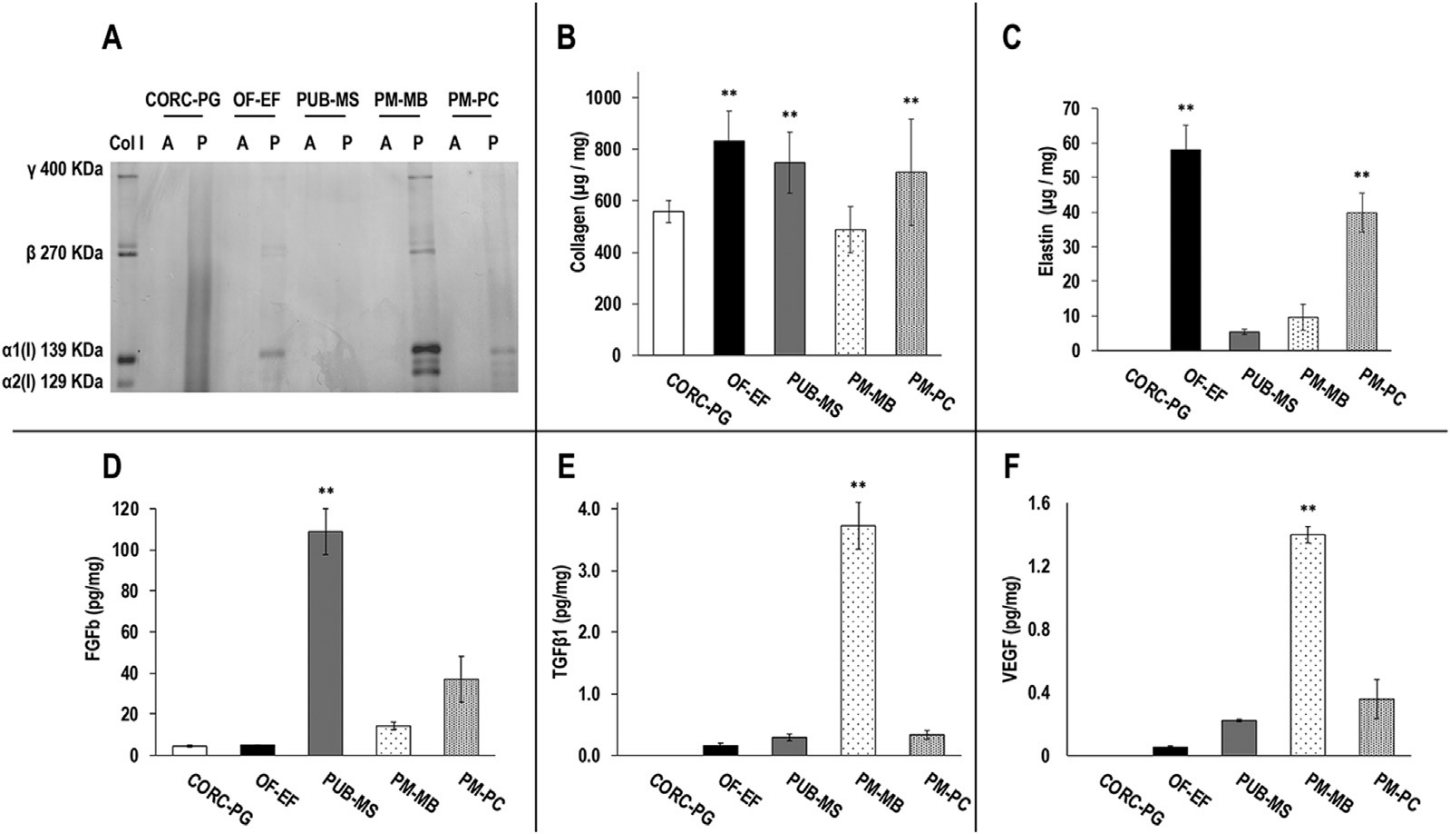
SDS-PAGE analysis of acetic acid (A) and acetic acid/pepsin (P) showed soluble collagen in OF-EF, PUB-MS, PM-MB, and PM-PC (A). Hydroxyproline assay revealed that the OF-EF, PUB-MS, and PM-PC had the highest (∗∗) collagen content (B). OF-EF and PM-PC showed the highest (∗∗) elastin content (C). The PUB-MS had the highest (∗∗) FGF-basic content (D). The PM-MB had the highest (∗∗) TGF-*β*1 (E) and VEGF (F) content. Data expressed as average ± standard deviation (n = 3). ∗∗Indicates statistically higher (*p*<0.01) groups.

SDS-PAGE analysis (Fig. 1A) revealed the presence of soluble collagen in OF-EF, PM-MB (highest amount), and PM-PC after acetic acid and pepsin extraction, whereas no soluble collagen was observed in any extraction of the CORC-PG and PUB-MS. Hydroxyproline assay (Fig. 1B) showed OF-EF, PUB-MS, and PM-PC to contain similar levels between them (*p*>0.05) and all of them showed significantly higher levels (*p*<0.01) than CORC-PG and PM-MB of collagen. OF-EF and PM-PC showed significantly higher (*p*<0.05) elastin content than PUB-MS and PM-MB, whereas no elastin was detected at the CORC-PG (Fig. 1C). PUB-MS exhibited the highest (*p*<0.001) amounts of FGF-basic (Fig. 1D). PM-MB had the highest (*p*<0.001) amounts of TGF-*β*1 (Fig. 1E) and VEGF (Fig. 1F). No growth factors were detected at the CORC-PG (Fig. 1D–F).

### 3.2 Histology and immunohistochemistry

**Fig. 2:**
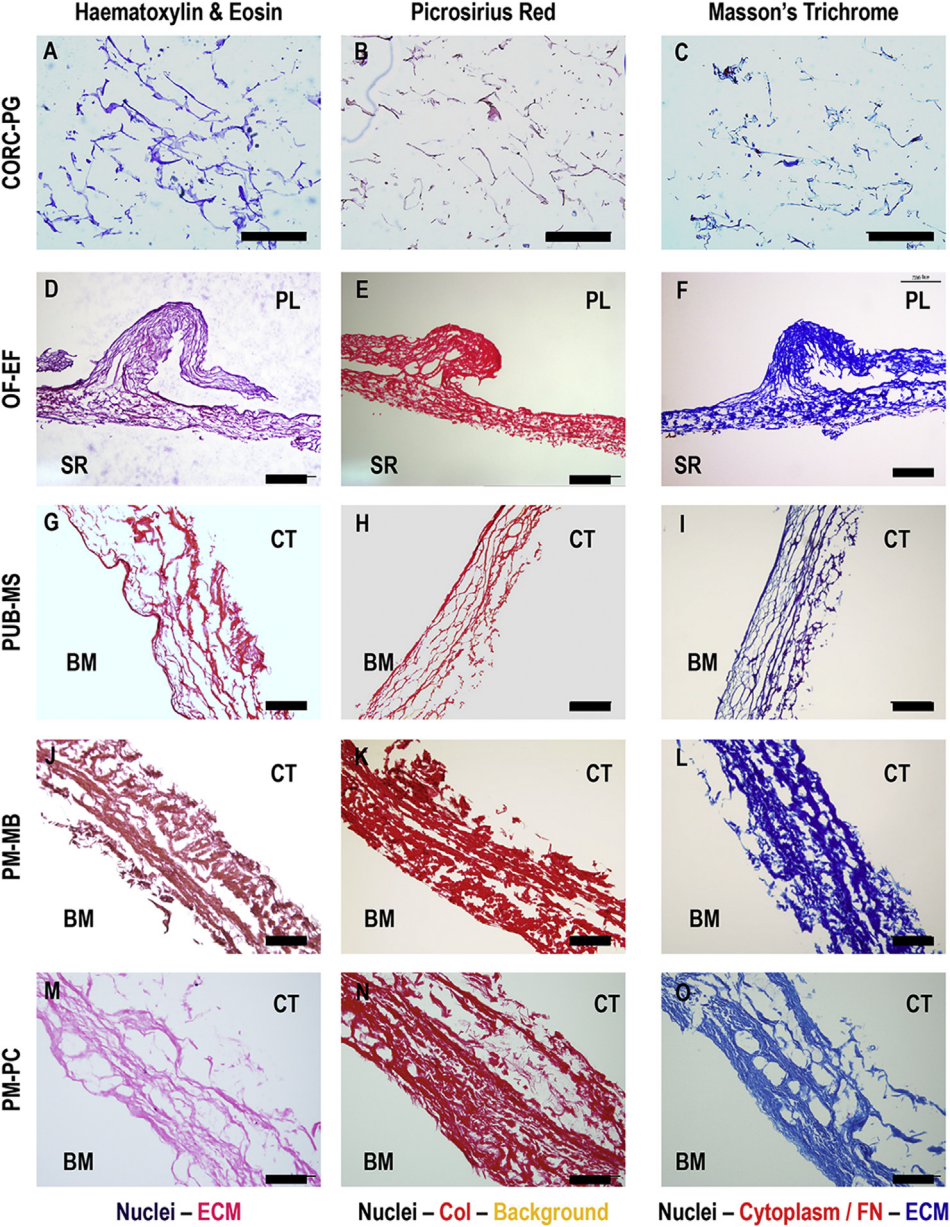
Histology analysis with hematoxylin/eosin, picrosirius red and Masson's trichrome of CORC-PG, OF-EF, PUB-MS, PM-MB, and PM-PC revealed a loose structure for the CORC-PG and a dense structure for the tissue graft materials. Scale bars 200 μm.

Histological analysis (Fig. 2) revealed a loose structure for the CORC-PG product (Fig. 2A–C), whereas all tissue grafts products exhibited a denser, tissue-like structure (Fig. 2D–O). Among the tissue grafts, the PUB-MS showed the least dense structure (Fig. 2G–I), whereas the OF-EF showed the highest preservation of tissue architecture (Fig. 2D–F). In PM-MB (Fig. 2J–L) and PM-PC (Fig. 2M–O), some cavities were observed, probably related to processing. Picrosirius red staining confirmed a dense collagenous network in all tissue grafts, especially in OF-EF (Fig. 2E), PM-MB (Fig. 2K), and PM-PC (Fig. 2N) products. Masson's trichrome staining revealed a red staining in OF-EF (Fig. 2F) and PUB-MS (Fig. 2I), which could correspond to residual cellular material or fibronectin.

**Fig. 3:**
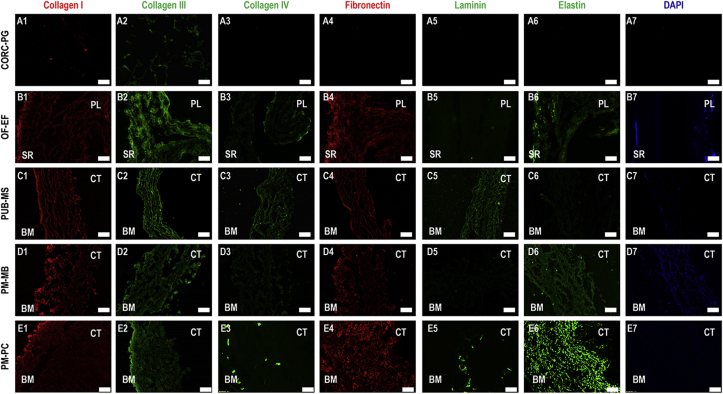
Immunohistochemistry analysis made apparent the presence of collagen type I, collagen type III, and fibronectin in all tissue grafts; collagen type IV in OF-EF, PUB-MS, and PM-PC; laminin in PUB-MS and PM-PC; and elastin in OF-EF, PM-MB, and PM-PC. Remaining cellular material was found in OF-EF and PM-MB. Scale bars 100 μm.

Immunohistochemistry analysis (Fig. 3) revealed the presence of collagen types I, III and fibronectin in all the tissue grafts products; collagen type IV was detected in OF-EF (Fig. 3B3), PUB-MS (Fig. 3C3), and PM-PC (Fig. 3E3); laminin was detected in PUB-MS (Fig. 3C5) and PM-PC (Fig. 3E5); and elastin was detected in PM-PC (Fig. 3E6), PM-MS (Fig. 3D6), and OF-EF (Fig. 3B6). DAPI-stained residual cellular material in OF-EF (Fig. 3B7), particularly in the SR side, in PM-MB (Fig. 3D7), and in some samples of PUB-MS, and PM-PC. CORC-PG showed very slight signals of collagen types I (Fig. 3A1) and III (Fig. 3A2) only.

### 3.3 Enzymatic degradation

**Fig. 4:**
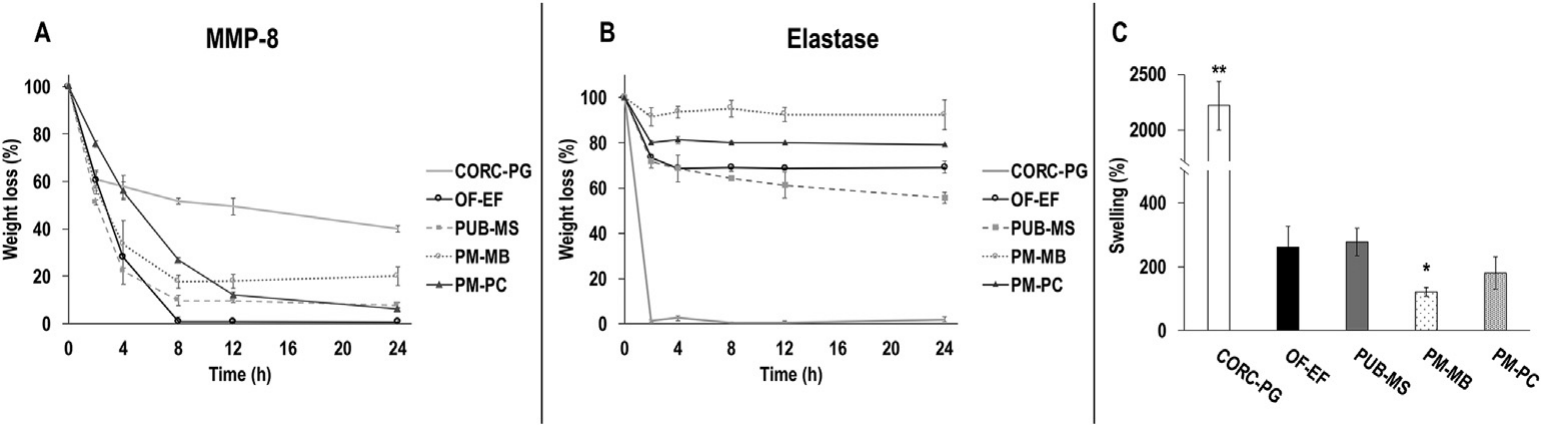
The CORC-PG showed the highest resistance to collagenase digestion (A) and the lowest resistance to elastase digestion (B). Among the tissue grafts, the PM-MB showed the highest resistance to collagenase (A) and elastase (B) digestion. The CORC-PG exhibited the highest PM-MB and the lowest swelling capacity (C). Data expressed as average ± standard deviation (n = 3). ∗∗Indicates statistically higher (*p*<0.01) groups and ∗Indicates statistically lower (*p<*0.05) groups.

The CORC-PG showed the highest resistance to collagenase degradation (*p*<0.05), whereas the PUB-MS, PM-MB, and PM-PC showed intermediate resistance and the OF-EF showed the lowest resistance to collagenase digestion (Fig. 4A). The CORC-PG showed the lowest resistance to elastase (*p<*0.05), whereas the OF-EF, PUB-MS, PM-MB, and PM-PC showed similar high resistance to elastase degradation (Fig. 4B).

### 3.4 Swelling analysis

Among the materials analyzed, the CORC-PG exhibited the highest (*p*<0.01) swelling capacity and the PM-MB showed the lowest (*p*<0.05) swelling capacity (Fig. 4C).

### 3.5 Bacterial penetration assay

**Fig. 5:**
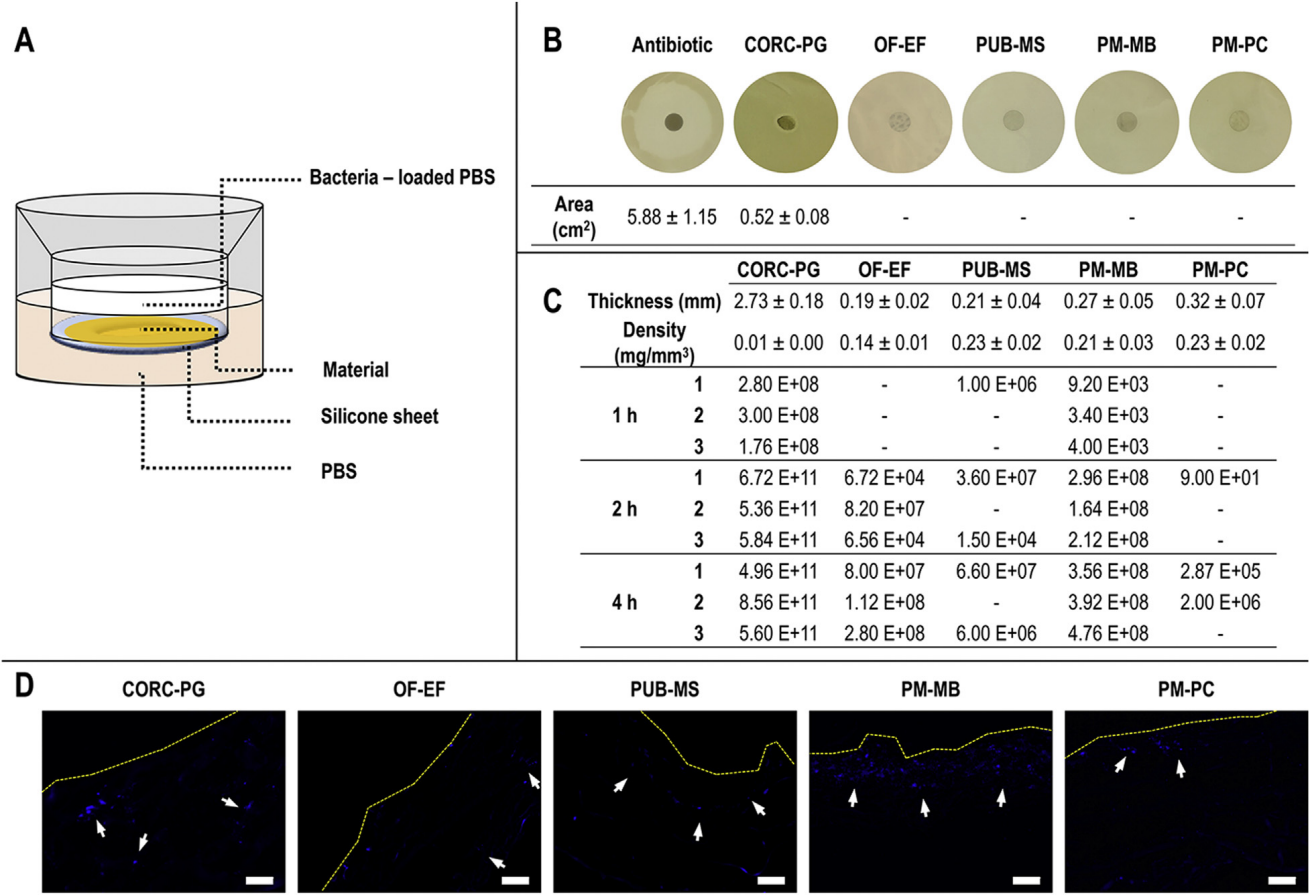
Bacterial penetration assay was carried out using an in-house trans-well system (A). Among the groups, only the CORC-PG showed bacteria growth inhibition (B). The CORC-PG showed the highest CFU number at all time points (C). Immunohistochemistry analysis of transverse sections after 24 h of bacterial incubation revealed bacterial colonization at the inner layers of the CORC-PG, OF-EF, and PUB-MS products (D). Area, thickness, and density data are expressed as average ± standard deviation (n = 4 for growth inhibition assay, n = 3 for thickness and density). Manual count of CFUs is represented with the value of each replicate; ‘-’ indicates the absence of colonies. Scale bars 50 μm.

Bacterial penetration was studied using an in-house developed trans-well system (Fig. 5A). From all products tested, only the positive antibiotic control and the CORC-PG showed bacteria growth inhibition (Fig. 5B). The CORC-PG showed the highest (*p*<0.05) CFU number at all times and the lowest (*p*<0.05) CFU number was detected for the OF-EF and PM-PC after 1 h, the PM-PC after 2 h, and PUB-MS and PM-PC after 4 h (Fig. 5C). Immunohistochemistry of transverse sections of the materials after 24 h of bacterial incubation showed accumulation of bacteria only at the interface with the PM-MB and PM-PC, whereas bacterial colonization at the inner layers of CORC-PG, OF-EF, and PUB-MS products was observed (Fig. 5D).

### 3.6 Dermal fibroblast response analysis

**Fig. 6:**
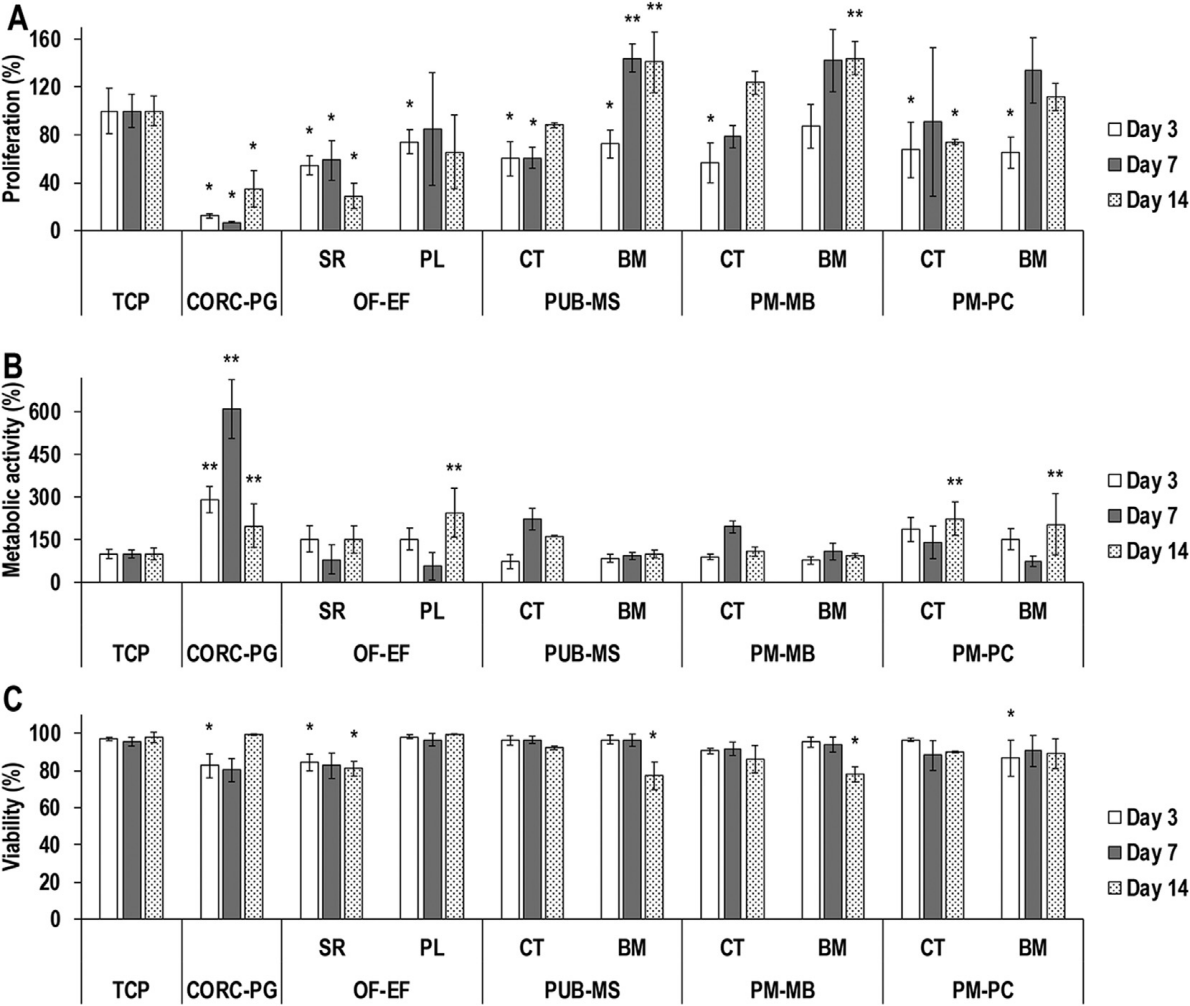
By day 14, the lowest (∗*p<*0.05) dermal fibroblast proliferation was detected for the CORC-PG, SR side of the OF-EF, and the CT side of the PM-PC, whereas the highest (∗∗*p<*0.05) dermal fibroblast proliferation was detected for the BM sides of the PUB-MS and PM-MB (A). By day 14, the CORC-PG, the PL side of the OF-EF, and both sides of PM-PC exhibited the highest (∗∗*p<*0.05) dermal fibroblast metabolic activity (B). By day 14, the SR side of the OF-EF, the BM side of the PUB-MS, and the MB side of the PM-MB showed the lowest (∗*p<*0.05) dermal fibroblast viability, although all groups exhibited viability higher than 75% (C). Data expressed as average ± standard deviation (n = 3). Samples were compared to the control TCP at a given time point.

In comparison to the control TCP, the lowest (*p<*0.05) dermal fibroblast proliferation (Fig. 6A and Supplementary Fig. S1) was detected for the CORC-PG, both sides of OF-EF and PUB-MS, CT side of PM-MB, and both sides of PM-PC at day 3; the CORC-PG, SR side of OF-EF, and CT side of PUB-MS at day 7; and the CORC-PG, SR side of OF-EF, and CT side of PM-PC at day 14. The highest (*p<*0.05) dermal fibroblast proliferation (Fig. 6A) was detected for the BM side of the PUB-MS at day 7 and the BM sides of the PUB-MS and PM-MB at day 14.

In comparison to the control TCP, the highest (*p<*0.05) dermal fibroblast metabolic activity (Fig. 6B) was detected for the CORC-PG at days 3 and 7, and the CORC-PG, PL side of OF-EF, and both sides of PM-PC at day 14.

In comparison to the control TCP, the lowest (*p<*0.05) dermal fibroblast viability (Fig. 6C and Supplementary Fig. S2) was detected for the CORC-PG, SR side of OF-EF, and BM side of PM-PC at day 3 and for the SR side of OF-EF, BM side of PUB-MS, and BM side of PM-MB at day 14.

### 3.7 Monocyte response analysis

**Fig. 7:**
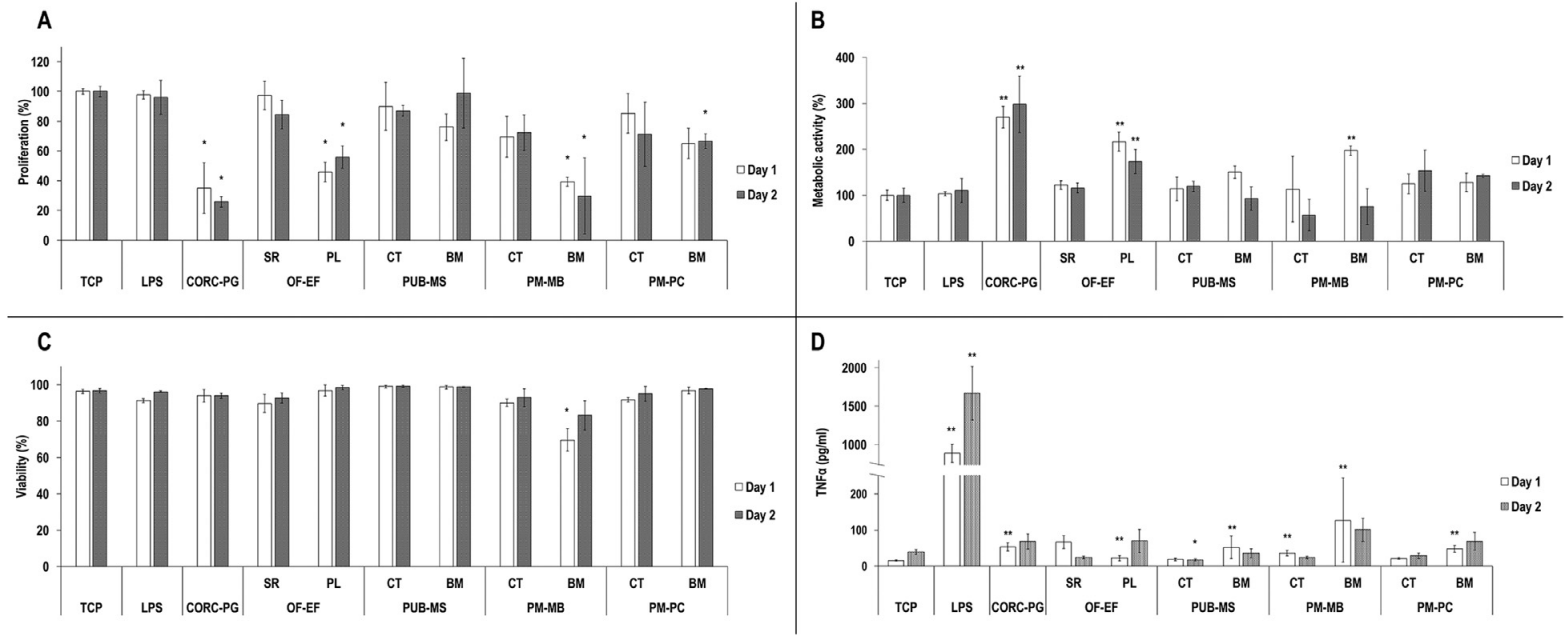
THP-1 response *in vitro* assessment revealed the lowest (∗*p*<0.05) proliferation on CORC-PG, PL side of OF-EF, and BM side of PM-MB at both time points (A). The highest (∗∗*p*<0.05) THP-1 metabolic activity was observed for the CORC-PG and the PL side of OF-EF at both time points (B). All groups exhibited similar (*p*>0.05) THP-1 viability at day 2 (C). Pro-inflammatory cytokine TNF-α analysis showed the highest (∗∗*p*<0.01) production by THP-1 cells in the LPS group (D). Among the test groups, higher TNF-α production (∗∗*p*<0.05) was observed on CORC-PG, PL side of OF-EF, and BM sides of PUB-PM, PM-MB, and PM-PC at day 1, although far from LPS levels (D). Data expressed as average ± standard deviation (n = 3). Samples were compared to the monocytes cultured on TCP with normal medium at a given time point.

In comparison to the control, the lowest (*p<*0.05) monocyte proliferation (Fig. 7A) was detected for the CORC-PG at days 1 and 2, PL side of the OF-EF at days 1 and 2, the BM side of PM-MB at days 1 and 2, and the BM side of PM-PC at day 2.

In comparison to the control, the highest (*p<*0.05) monocyte metabolic activity (Fig. 7B) was detected for the CORC-PG at days 1 and 2, the PL side of the OF-EF at days 1 and 2, and the BM side of PM-MB at day 1. In comparison to the control, the lowest (*p<*0.05) monocyte viability (Fig. 7C and Supplementary Fig. S3) was detected for the BM side of PM-MB at day 1. In comparison to the control, the highest (*p<*0.01) TNF-α production (Fig. 7D) was observed for the LPS group at days 1 and 2. Furthermore, in comparison to the control, the lowest (*p*<0.05) TNF-α production (Fig. 7D) was detected for the CT side of PUB-MS at day 2; whereas the highest (*p<*0.05) TNF-α production (Fig. 7D) was observed for the CORC-PG at day 1, the PL side of the OF-EF at day 1, the BM side of the PUB-MS at day 1, the BM side of the PM-MB at day 1, and the BM side of PM-PC at day 1.

When THP-1 were treated with the materials' conditioned media, in comparison to the control, the highest (*p*<0.05) proliferation at day 1 was detected for the CORC-PG, OF-EF, PM-MB, and PM-PC, and no differences were observed between the groups at day 2 (Supplementary Fig. S4A). In comparison to the control, the lowest (*p*<0.05) THP-1 metabolic activity was found when they were treated with conditioned media of CORC-PG at day 1 and with conditioned media of PUB-MS, PM-MB, and PM-PC at day 2 (Supplementary Fig. S4B). In comparison to the control, the conditioned media of all materials induced significantly lower *(p*<0.05) THP-1 viability at day 2 (Supplementary Fig. S4C). TNF-α production by LPS was the highest (*p*<0.01) at days 1 and 2, and no significant differences (*p*>0.05) were observed between the control and THP-1 cells treated with any of the materials’ conditioned media (Supplementary Fig. S4D).

**Fig. 8:**
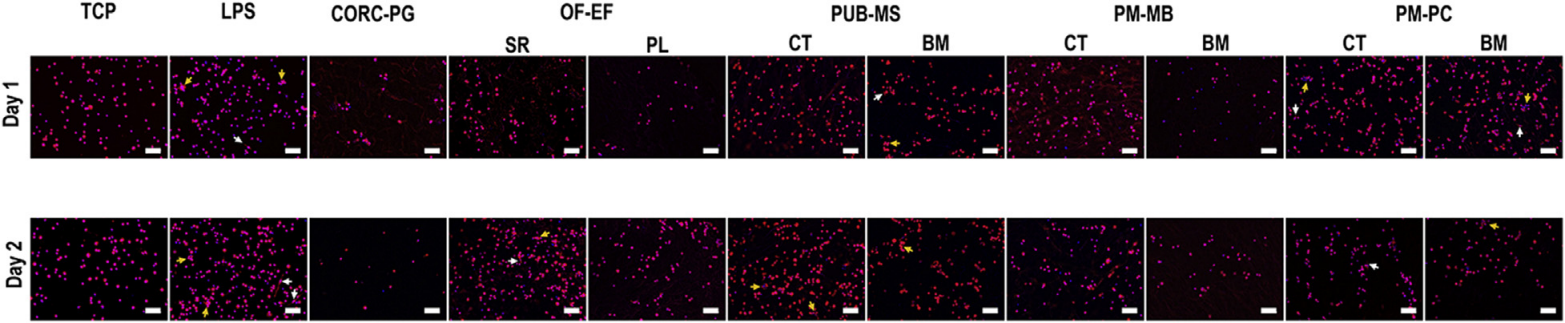
Immunocytochemistry (red: cytoskeleton; blue: nuclei) analysis of THP-1 revealed the presence of elongated cells (white arrows; LPS at days 1 and 2, SR side of OF-EF at day 2, BM side of PUB-MS at day 1, CT side of PM-PC at days 1 and 2, and BM side of PM-PC at day 1) and cell clusters (yellow arrows; LPS at days 1 and 2, SR side of OF-EF at day 2, BM side of PUB-MS at day 1 and both sides at day 2, both sides of PM-PC at day 1, and BM side of PM-PC at day 2). Scale bars 100 μm.

Immunocytochemistry analysis of the cytoskeleton revealed that all treatments resulted in rounded cell morphology, although some elongated cells were detected in the LPS (days 1 and 2), SR side of OF-EF at day 2, BM side of PUB-MS at day 1, CT side of PM-PC at days 1 and 2, and BM side of PM-PC at day 1. Some cell clusters (>5 cells) were also observed in the LPS (days 1 and 2), SR side of OF-EF at day 2, BM side of PUB-MS at day 1, CT and BM sides at day 2 of PUB-MS, CT and BM sides of PM-PC at day 1, and BM side of PM-PC at day 2 (Fig. 8).

### 3.8 Scratch and rat aortic ring assays

**Fig. 9:**
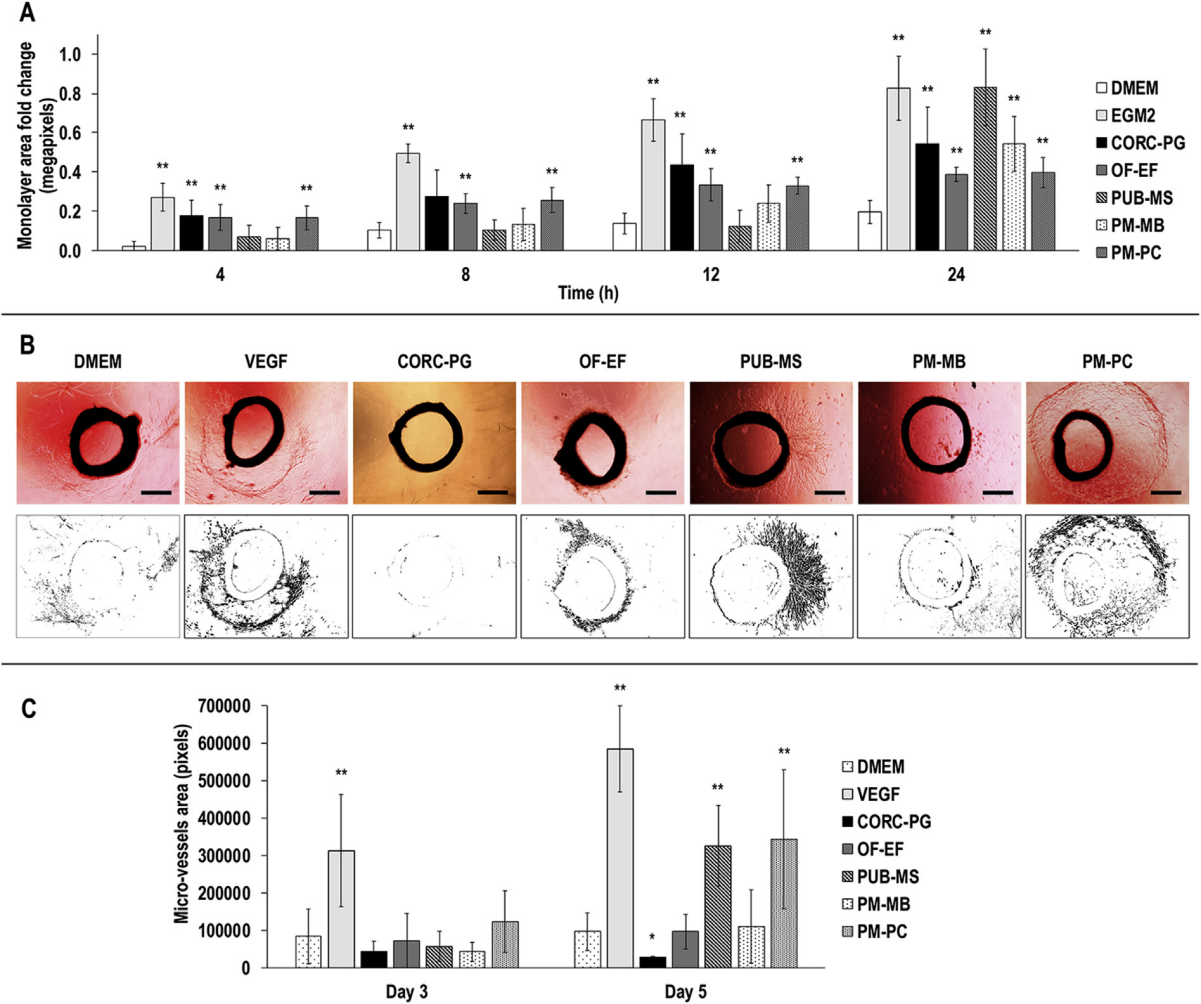
HUVECs scratch assay analysis revealed that after 24 h, all groups demonstrated significantly higher (∗∗) monolayer area fold change (A). Representative images and binary masks of aortic rings at day 5 showed formation of micro-vessels in all conditions except CORC-PG (B). At day 5, the VEGF, PUB-MS, and PM-PC showed the highest (∗∗) and the CORC-PG the lowest (∗) micro-vessels area quantification (C). Scale bars 50 μm. Data are expressed as average ± standard deviation (n = 5 for scratch assay, n = 3 for aortic ring assay). Statistically significant: *p*<0.05.

In comparison to DMEM control, the EGM2 was significantly higher (*p<*0.01) in monolayer area fold change at all time points (Fig. 9A, Supplementary Fig. S5). Furthermore, in comparison to the DMEM control, the highest (*p<*0.05) monolayer area fold change was observed for the CORC-PG, OF-EF, and PM-PC groups at 4 h, the OF-EF and PM-PC groups at 8 h, the CORC-PG, OF-EF, and PM-PC groups at 12 h, and all the groups at 24 h (Fig. 9A, Supplementary Fig. S5).

Microscopy analysis of sectioned aorta rings revealed that only the CORC-PG group was not able to produce micro-vessels and also resulted in media discoloration, indicative of low pH (Fig. 9B).

Micro-vessel quantification (Fig. 9C) revealed that, in comparison to DMEM control, VEGF micro-vessel area was significantly higher (*p*<0.05) at days 3 and 5. Furthermore, in comparison to DMEM control, the lowest (*p<*0.05) micro-vessel area was found in CORC-PG at day 5, whereas the highest (*p*<0.05) micro-vessel area was observed in PUB-MS and PM-PC groups at day 5.

## 4 Discussion

Although porcine mesothelium has structural, compositional, and biological properties potentially beneficial for wound healing applications [[15], [16], [17],^21^,^22^], to-date, it has been used only for breast [13], cartilage [26], and nasal [27] reconstruction and as tendon protector sheet [21]. To assess whether porcine mesothelium grafts are indeed good candidates for wound healing applications, herein, we compared the properties of the only two commercially available decellularized porcine mesothelium xenografts (PM-MB and PM-PC) to traditionally used wound healing xenografts (OF-EF and PUB-MS) and biomaterials (CORC-PG). In addition to the different tissue sources, the tissue grafts used herein were also processed differently, which also impacts on their features and biological response. For instance, OF-EF and PM-PC are processed with detergents and osmotic solutions and are sterilized with ethylene oxide and gamma irradiation, respectively. The PUB-MS is processed with peracetic acid and ethanol and sterilized with electron beam irradiation. The PM-MS is processed using the OPTRIX™ Tissue Process protocol, which gently disinfects tissues, inactivates viruses, removes cells, and retains native tissue composition. However, the details of the individual processing conditions remain confidential/trade secret and as such their correlation to their final characteristics is elusive.

SDS-PAGE of acid/pepsin treated materials revealed soluble collagen only in PM-MB, PM-PC, and OF-EF materials, whereas hydroxyproline assay revealed that all materials were comprised of collagen. Elastin quantification revealed no elastin in CORC-PG biomaterial, which is not surprising considering that it is produced from extracted collagen, and among the tissue grafts, the OF-EF and the PM-PC had the highest elastin content. The observed differences among the various grafts can be attributed to the different processing [[41], [42], [43]] and cross-linking density, which is species, age, and tissue dependent [20,[44], [45], [46], [47], [48], [49]].

Growth factors such as FGF-basic, VEGF, and TGF-*β*1 can be retained in the ECM after decellularization [19,[50], [51], [52]] and are known to promote key events in would healing, such as cell proliferation and migration and angiogenesis [24,53]. In this study, all three growth factors were detected in the porcine grafts, as has been reported previously [22,54]. OF-EF also preserved VEGF and TGF-*β*1, but to a lower extent than the porcine materials. Previous work has shown OF-EF to contain FGF-basic, but in amounts below the background detection of this study [55]. On the other hand, PM-MB contained higher amounts of TGF-*β*1 and VEGF than the rest of the materials tested, which could enhance wound healing events *in vivo*. However, high levels TGF-*β*1 could also trigger fibrosis events [56]. Such differences in growth factor content among the products may be attributed to their diverse range of tissue and/or processing. As expected, growth factors were undetected in CORC-PG biomaterial.

Histology and immunohistochemistry analyses confirmed the maintenance of tissue structure and presence of fundamental components (e.g. collagen types I and III, fibronectin) in the ECM products, as it has been reported previously [16,17,22,55,57]. The CORC-PG biomaterial exhibited a loose, sponge-like structure, as it has been described before [58], largely attributed to its lyophilization manufacturing process. Tissue grafts presented a laminar and fibrous structure, closely imitating the native (pre-processing) tissue structure. Decellularization protocols and further processing can affect the structure, integrity, and composition of tissue grafts [22,59], which explains the observed differences in tissue preservation, as revealed by histology and immunohistochemistry analyses among the different tissue graft products. The presence of elastin was much more intense in the PM-PC compared to PM-MB and PUB-MS, matching the colorimetric quantification results, conversely to OF-EF, which staining was expected to be more intense. Such differences could be due to the specificity of the immunohistochemistry antibody between species, considering that the presence of elastin in OF-EF has been previously documented [55]. DAPI staining revealed residual cellular material in OF-EF and PM-MB products, which has been related to immune reactions *in vivo* [60].

MMPs play a crucial role in the wound healing [61] and their modulation is a desirable and characteristic feature of collagen-based biomaterials [62]. CORC-PG showed the highest resistance to MMP-8 degradation; however, the collagen component of this material (55%) was completely degraded within the first 4 h, indicating that the cellulose component (45%) was responsible for the resistance to enzymatic degradation and that collagen acted as a sacrificial substrate [63], which would decrease the activity of MMPs in the wound environment [64]. The resistance to porcine pancreatic elastase, which keeps similar substrate specificity with human neutrophil elastase [65], was also assessed. The CORC-PG biomaterial was completely degraded in 2 h, as has been observed in previous studies [63], where CORC-PG acted as substrate for neutrophil elastase, thereby reducing its activity in wound fluid. The tissue grafts showed a proportional resistance to enzymatic degradation; less than 20% remained after 24 h of MMP-8 incubation and more than 60% remained after 24 h elastase incubation, both of which can be explained considering their compositional analysis. Observed differences among them could be attributed to their heterogeneous composition, donor variability, and processing. In any case, their higher resistance to proteolytic degradation than collagen-based biomaterials could translate to a slower resorption rate and the need of fewer applications, which would ultimately reduce healthcare costs [3].

Exudates uptake from the wound and maintenance of the appropriate moisture is a desirable characteristic of a wound dressing [66]. The CORC-PG biomaterials swelled the most because of its rather ‘simple’ (in comparison to the tissue grafts) composition and highly-porous structure (in comparison to the laminar, fibrous, and less porous structure of the tissue grafts), as observed in this study by histological analysis and in previous studies with scanning electron microscopy analysis of CORC-PG [58], OF-EF [55], PUB-MS [59,67], PM-MB [68], and PM-PC [21]. PM-MB exhibited the lowest swelling, probably due to differences in structure, processing, and/or crosslinking density [69].

Infection is a major complication in wound healing [70], where ECM products have been shown to be an effective alternative to synthetic materials [10,71]. In a wound healing scenario, tissue grafts and biomaterials applied on the wound become the only barrier to pathogen penetration into the wound when it is exposed (e.g. during re-application) [72]. We therefore used a model to evaluate the potential of these materials as microbial barrier, based on previous studies [38,39]. It is worth noting that although this *in vitro* model does not recapitulate the *in vivo* bacterial penetration in a wound setting (i.e. higher pathogen concentration, longer exposure time), it can act as an effective screening tool for material selection/screening to proceed to preclinical assessment. As per previous reports [73,74], in the absence of any antibiotic, none of the tissue grafts inhibited bacterial growth, considering that collagen type IV, fibronectin, and laminin have been shown to bind and aggregate bacteria [75]. The CORC-PG presented a slight bacterial inhibition, which has been previously attributed to the oxidized regenerated cellulose component [76]; however, its porous structure allowed immediate bacterial invasion. Among the tissue grafts, the PM-PC showed the lowest bacteria colonization/penetration capacity, possibly attributed to its denser structure. This is in accordance with previous publications where lower porosity and/or BM preservation have been shown to inhibit bacterial colonization/penetration [39,77].

Cytocompatibility analysis with dermal fibroblasts showed that all tissue grafts were capable of supporting cell growth, as has been observed in previous studies [[21], [22], [23]]. Although some statistically significant differences were observed between the groups, all exhibited >75% dermal fibroblast viability, which, in general, is not considered as biologically significant. The PL and the BM sides of the grafts showed higher cell growth than the SR and the connective sides, respectively, largely attributed to higher amounts of collagen type IV, laminin, and/or fibronectin that have been shown to promote cellular growth [[78], [79], [80]], albeit with variable degree of cell specificity [81] and attachment and spreading [82]. Cells on CORC-PG showed the lowest proliferation and the highest metabolic activity among the tested samples, which could be due to the fast loss of the collagen and lower cytocompatibility of the oxidized regenerated cellulose. In fact, CORC-PG has previously been shown to support the attachment of 3T3 fibroblasts, but with limited growth [58].

The CORC-PG, the PL side of OF-EF, and the BM side of the PM-MB and PM-PC showed the lowest proliferation and the CORC-PG and PL side of OF-EF showed the highest metabolic activity at both time points when seeded with THP-1, which could be related to an inflammatory response [21]. However, for all materials, the production of TNF-*α* was far lower from the levels observed in LPS group, which matches previous studies with cellulose scaffolds [83], urinary bladder matrix [57,84], and porcine mesothelium [21]. Although some elongated cells and cell clusters were observed in some conditions, we cannot conclusively corelate these observations with THP-1 polarization, as cell morphology of differentiated THP-1 can be influenced by the surface (e.g. topographical features) and bulk (e.g. substrate elasticity) properties of the under investigation scaffolds [85,86]. However, TNF-α analysis indicated M2 or combined M1/M2 polarization, as per previous studies of decellularized grafts [57,84], which could promote remodeling *in vivo*. The higher TNF-*α* production in the BM side, as opposed to the CT side, of the porcine grafts may be related to their higher laminin and fibronectin content [15,[87], [88], [89], [90], [91]]. When cells were treated with materials' conditioned media, no particular differences were observed, indicating that the effects observed in direct contact are not related to materials’ soluble factors and degradation products.

In the scratch test, all materials promoted higher migration than the negative control after 24 h. In the case of CORC-PG, this phenomenon may be due to the solubilization of the collagenous fraction, which has been shown to promote cell migration of endothelial cells [92,93]. The observed high migration of the tissue grafts may be attributed to the release of soluble factors that promote angiogenesis, such as FGF-basic, TGF-*β*1, and VEGF [24,53]. The aortic ring assay showed only the PUB-MS and PM-PC products to promote micro-vessel formation when compared to DMEM control, whereas the CORC-PG did not allow the formation of micro-vessels. This is in contrast to previous studies, where OF-EF showed an angiogenic effect on rat aorta rings [40], which may account for batch-to-batch variability or differences in the protocol, because in this study, the FBS may have masked the effect on the angiogenesis of OF-EF [94]. Also, despite the higher presence of VEGF and TGF-*β*1 in PM-MB, the higher total amount and combination of growth factors in PUB-MS and PM-PC could have triggered a higher synergistic effect on the angiogenesis [95]. Nonetheless, these results provide evidence for the angiogenic effect of porcine mesothelium tissues similarly to the porcine urinary bladder that its angiogenic effect has been reported previously [96].

## 5 Conclusion

Xenografts are extensively used in biomedicine because of their abundant availability, structural features, biochemical composition, and biological properties. Herein, we demonstrated that porcine mesothelium grafts (e.g. PM-MB and PM-PC), although previously not designed for wound healing applications, they have similar or even superior properties to traditionally used wound healing xenografts (e.g. Endoform™ and MatriStem®) and biomaterials (e.g. Promogran™). Considering that there is no widely accepted tissue graft or biomaterial therapy for wound healing applications, this study paves the way for diversification of already clinically available materials, thus reducing the timeframe to bedside.

## Author contribution

Héctor Capella-Monsonís contributed to conceptualisation, methodology, data analysis, writing first draft and subsequent reviewing and editing. Maura A. Tilbury and J. Gerard Wall contributed to microbial analysis and manuscript reviewing and editing. Dimitrios I. Zeugolis contributed to conceptualisation, methodology, supervision and manuscript writing, reviewing and editing. All authors have approved the manuscript.

## Conflict of interest

The authors declare that they have no known competing financial interests or personal relationships that could have appeared to influence the work reported in this paper.

## References

[1] Gould L.J. (2016). Topical collagen-based biomaterials for chronic wounds: rationale and clinical application. Adv. Wound Care.

[2] Zielins E.R., Atashroo D.A., Maan Z.N., Duscher D., Walmsley G.G., Hu M., Senarath-Yapa K., McArdle A., Tevlin R., Wearda T., Paik K.J., Duldulao C., Hong W.X., Gurtner G.C., Longaker M.T. (2014). Wound healing: an update. Regen. Med..

[3] Lindholm C., Searle R. (2016). Wound management for the 21st century: combining effectiveness and efficiency. Int. Wound J..

[4] Mostow E.N., Haraway G.D., Dalsing M., Hodde J.P., King D. (2005). Effectiveness of an extracellular matrix graft (OASIS Wound Matrix) in the treatment of chronic leg ulcers: a randomized clinical trial. J. Vasc. Surg..

[5] Martinson M., Martinson N. (2016). A comparative analysis of skin substitutes used in the management of diabetic foot ulcers. J. Wound Care.

[6] Romanelli M., Dini V., Bertone M., Barbanera S., Brilli C. (2007). OASIS® wound matrix versus Hyaloskin® in the treatment of difficult-to-heal wounds of mixed arterial/venous aetiology. Int. Wound J..

[7] LeCheminant J., Field C. (2012). Porcine urinary bladder matrix: a retrospective study and establishment of protocol. J. Wound Care.

[8] Burkey B., Davis W., Glat P.M. (2016). Porcine xenograft treatment of superficial partial-thickness burns in paediatric patients. J. Wound Care.

[9] Catena F., Ansaloni L., Gazzotti F., Gagliardi S., Di Saverio S., D'Alessandro L., Pinna A.D. (2007). Use of porcine dermal collagen graft (Permacol) for hernia repair in contaminated fields. Hernia.

[10] Abdelfatah M.M., Rostambeigi N., Podgaetz E., Sarr M.G. (2015). Long-term outcomes (>5-year follow-up) with porcine acellular dermal matrix (Permacol™) in incisional hernias at risk for infection. Hernia.

[11] Adeel A., Tyler B., Brian R. (2017). Repair of complete atrioventricular septal defects with decellularized extracellular matrix: initial and midterm outcomes. World J. Pediatr. Congenit. Heart Surg..

[12] Kelley T.M., Kashem M., Wang H., McCarthy J., Carroll N.D., Moser G.W., Guy T.S. (2017). Anterior leaflet augmentation with CorMatrix porcine extracellular matrix in twenty-five patients: unexpected patch failures and histologic analysis. Ann. Thorac. Surg..

[13] Hillberg N.S., Ferdinandus P.I., Dikmans R.E.G., Winkens B., Hommes J., van der Hulst R.R.W.J. (2018). Is single-stage implant-based breast reconstruction (SSBR) with an acellular matrix safe?: Strattice™ or Meso Biomatrix® in SSBR. Eur. J. Plast. Surg..

[14] Salzberg C.A., Dunavant C., Nocera N. (2013). Immediate breast reconstruction using porcine acellular dermal matrix (Strattice®): long-term outcomes and complications. J. Plast. Reconstr. Aesthetic Surg..

[15] Blackburn S.C., Stanton M.P. (2014). Anatomy and physiology of the peritoneum. Semin. Pediatr. Surg..

[16] Witz C.A., Montoya-Rodriguez I.A., Cho S., Centonze V.E., Bonewald L.F., Schenken R.S. (2001). Composition of the extracellular matrix of the peritoneum. J. Soc. Gynecol. Invest..

[17] Jasna T., Nesic D., Laušević Z., Miljana O., Goran B., Biljana S. (2006). Histological characteristics of healthy animal peritoneum. Acta Vet..

[18] Taylor D.A., Sampaio L.C., Ferdous Z., Gobin A.S., Taite L.J. (2018). Decellularized matrices in regenerative medicine. Acta Biomater..

[19] Nowocin A.K., Southgate A., Gabe S.M., Ansari T. (2016). Biocompatibility and potential of decellularized porcine small intestine to support cellular attachment and growth. J. Tissue Eng. Regen. Med..

[20] Sorushanova A., Delgado L.M., Wu Z. (2019). The collagen suprafamily: from biosynthesis to advanced biomaterial development. Adv. Mater..

[21] Capella-Monsonis H., Kelly J., Kearns S., Zeugolis D.I. (2019). Decellularised porcine peritoneum as a tendon protector sheet. Biomed. Mater..

[22] Hoganson D.M., Owens G.E., O'Doherty E.M., Bowley C.M., Goldman S.M., Harilal D.O., Neville C.M., Kronengold R.T., Vacanti J.P. (2010). Preserved extracellular matrix components and retained biological activity in decellularized porcine mesothelium. Biomaterials.

[23] Tsai P.C., Zhang Z., Florek C., Michniak-Kohn B.B. (2016). Constructing human skin equivalents on porcine acellular peritoneum extracellular matrix for in vitro irritation testing. Tissue Eng. A.

[24] Werner S., Grose R. (2003). Regulation of wound healing by growth factors and cytokines. Physiol. Rev..

[25] Tracy L.E., Minasian R.A., Caterson E.J. (2016). Extracellular matrix and dermal fibroblast function in the healing wound. Adv. Wound Care.

[26] Meng Q., Hu X., Huang H., Liu Z., Yuan L., Shao Z., Jiang Y., Zhang J., Fu X., Duan X., Ao Y. (2017). Microfracture combined with functional pig peritoneum-derived acellular matrix for cartilage repair in rabbit models. Acta Biomater..

[27] Bramos A., Perrault D.P., Fedenko A.N., Kim G.H., Bougioukli S., Lieberman J.R., Calvert J.W., Wong A.K. (2018). Porcine mesothelium-wrapped diced cartilage grafts for nasal reconstruction. Tissue Eng..

[28] Liden B.A., May B.C.H. (2013). Clinical outcomes following the use of ovine forestomach matrix (Endoform Dermal Template) to treat chronic wounds. Adv. Skin Wound Care.

[29] Kim J.S., Kaminsky A.J., Summitt J.B., Thayer W.P. (2016). New innovations for deep partial-thickness burn treatment with ACell MatriStem Matrix. Adv. Wound Care.

[30] Frykberg R.G., Cazzell S.M., Arroyo-Rivera J., Tallis A., Reyzelman A.M., Saba F., Warren L., Stouch B.C., Gilbert T.W. (2016). Evaluation of tissue engineering products for the management of neuropathic diabetic foot ulcers: an interim analysis. J. Wound Care.

[31] Lobmann R., Zemlin C., Motzkau M., Reschke K., Lehnert H. (2006). Expression of matrix metalloproteinases and growth factors in diabetic foot wounds treated with a protease absorbent dressing. J. Diabetes Complicat..

[32] Veves A., Sheehan P., Pham H.T. (2002). For the Promogran Diabetic Foot Ulcer, A randomized, controlled trial of promogran (a collagen/oxidized regenerated cellulose dressing) vs standard treatment in the management of diabetic foot ulcers. Arch. Surg..

[33] Ghatnekar O., Willis M., Persson U. (2002). Cost-effectiveness of treating deep diabetic foot ulcers with Promogran in four European countries. J. Wound Care.

[34] Kakagia D.D., Kazakos K.J., Xarchas K.C., Karanikas M., Georgiadis G.S., Tripsiannis G., Manolas C. (2007). Synergistic action of protease-modulating matrix and autologous growth factors in healing of diabetic foot ulcers. A prospective randomized trial. J. Diabetes Complicat..

[35] Capella-Monsonis H., Coentro J.Q., Graceffa V., Wu Z., Zeugolis D.I. (2018). An experimental toolbox for characterization of mammalian collagen type I in biological specimens. Nat. Protoc..

[36] Satyam A., Kumar P., Fan X., Gorelov A., Rochev Y., Joshi L., Peinado H., Lyden D., Thomas B., Rodriguez B., Raghunath M., Pandit A., Zeugolis D. (2014). Macromolecular crowding meets tissue engineering by self-assembly: a paradigm shift in regenerative medicine. Adv. Mater..

[37] Lu Q., Ganesan K., Simionescu D.T., Vyavahare N.R. (2004). Novel porous aortic elastin and collagen scaffolds for tissue engineering. Biomaterials.

[38] Woo C.H., Choi Y.C., Choi J.S., Lee H.Y., Cho Y.W. (2015). A bilayer composite composed of TiO2-incorporated electrospun chitosan membrane and human extracellular matrix sheet as a wound dressing. J. Biomater. Sci. Polym. Ed..

[39] Yaghobee S., Samadi N., Khorsand A., Ghahroudi Amir Ali R., Kadkhodazadeh M. (2014). Comparison of the penetration and passage of Streptococcus mutans and Aggregatibacter actinomycetemcomitans through membranes loaded with tetracycline, amoxicillin, and chlorhexidine: an in vitro study. J. Basic Clin. Physiol. Pharmacol..

[40] Irvine S.M., Cayzer J., Todd E.M., Lun S., Floden E.W., Negron L., Fisher J.N., Dempsey S.G., Alexander A., Hill M.C., O'Rouke A., Gunningham S.P., Knight C., Davis P.F., Ward B.R., May B.C.H. (2011). Quantification of in vitro and in vivo angiogenesis stimulated by ovine forestomach matrix biomaterial. Biomaterials.

[41] Schenke-Layland K., Vasilevski O., Opitz F., König K., Riemann I., Halbhuber K.J., Wahlers T., Stock U.A. (2003). Impact of decellularization of xenogeneic tissue on extracellular matrix integrity for tissue engineering of heart valves. J. Struct. Biol..

[42] Partington L., Mordan N.J., Mason C., Knowles J.C., Kim H.W., Lowdell M.W., Birchall M.A., Wall I.B. (2013). Biochemical changes caused by decellularization may compromise mechanical integrity of tracheal scaffolds. Acta Biomater..

[43] Gilbert T.W., Sellaro T.L., Badylak S.F. (2006). Decellularization of tissues and organs. Biomaterials.

[44] Debeer S., Le Luduec J.-B., Kaiserlian D., Laurent P., Nicolas J.-F., Dubois B., Kanitakis J. (2013). Comparative histology and immunohistochemistry of porcine versus human skin. Eur. J. Dermatol..

[45] Kurtz A., Oh S.-J. (2012). Age related changes of the extracellular matrix and stem cell maintenance. Prev. Med..

[46] Hosoda Y., Kawano K., Yamasawa F., Ishii T., Shibata T., Inayama S. (1984). Age-dependent changes of collagen and elastin content in human aorta and pulmonary artery. Angiology.

[47] Cannon D.J., Davison P.F. (1977). Aging, and crosslinking in mammalian collagen. Exp. Aging Res..

[48] Reddy N., Reddy R., Jiang Q. (2015). Crosslinking biopolymers for biomedical applications. Trends Biotechnol..

[49] Walters B.D., Stegemann J.P. (2014). Strategies for directing the structure and function of 3D collagen biomaterials across length scales. Acta Biomater..

[50] Erdbrugger W., Konertz W., Dohmen P.M., Posner S., Ellerbrok H., Brodde O.E., Robenek H., Modersohn D., Pruss A., Holinski S., Stein-Konertz M., Pauli G. (2006). Decellularized xenogenic heart valves reveal remodeling and growth potential in vivo. Tissue Eng..

[51] Hodde J.P., Ernst D.M.J., Hiles M.C. (2005). An investigation of the long-term bioactivity of endogenous growth factor in OASIS Wound Matrix. J. Wound Care.

[52] McDevitt C.A., Wildey G.M., Cutrone R.M. (2003). Transforming growth factor-β1 in a sterilized tissue derived from the pig small intestine submucosa. J. Biomed. Mater. Res. A.

[53] Behm B., Babilas P., Landthaler M., Schreml S. (2012). Cytokines, chemokines and growth factors in wound healing. J. Eur. Acad. Dermatol. Venereol..

[54] Chun S.Y., Lim G.J., Kwon T.G., Kwak E.K., Kim B.W., Atala A., Yoo J.J. (2007). Identification and characterization of bioactive factors in bladder submucosa matrix. Biomaterials.

[55] Lun S., Irvine S.M., Johnson K.D., Fisher N.J., Floden E.W., Negron L., Dempsey S.G., McLaughlin R.J., Vasudevamurthy M., Ward B.R., May B.C.H. (2010). A functional extracellular matrix biomaterial derived from ovine forestomach. Biomaterials.

[56] Coentro J.Q., Pugliese E., Hanley G., Raghunath M., Zeugolis D.I. (2018). Current and Upcoming Therapies to Modulate Skin Scarring and Fibrosis, Advanced Drug Delivery Reviews.

[57] Sadtler K., Sommerfeld S.D., Wolf M.T., Wang X., Majumdar S., Chung L., Kelkar D.S., Pandey A., Elisseeff J.H. (2017). Proteomic composition and immunomodulatory properties of urinary bladder matrix scaffolds in homeostasis and injury. Semin. Immunol..

[58] Karr J.C., Taddei A.R., Picchietti S., Gambellini G., Fausto A.M., Giorgi F. (2011). A morphological and biochemical analysis comparative study of the collagen products Biopad, Promogram, Puracol, and Colactive. Adv. Skin Wound Care.

[59] Faulk D.M., Carruthers C.A., Warner H.J., Kramer C.R., Reing J.E., Zhang L., D'Amore A., Badylak S.F. (2014). The effect of detergents on the basement membrane complex of a biologic scaffold material. Acta Biomater..

[60] Keane T.J., Londono R., Turner N.J., Badylak S.F. (2012). Consequences of ineffective decellularization of biologic scaffolds on the host response. Biomaterials.

[61] Rohani M.G., Parks W.C. (2015). Matrix remodeling by MMPs during wound repair. Matrix Biol..

[62] Chattopadhyay S., Raines R.T. (2014). Collagen-based biomaterials for wound healing. Biopolymers.

[63] Cullen B., Smith R., McCulloch E., Silcock D., Morrison L. (2002). Mechanism of action of PROMOGRAN, a protease modulating matrix, for the treatment of diabetic foot ulcers. Wound Repair Regen..

[64] Wu S., Applewhite A.J., Niezgoda J., Snyder R., Shah J., Cullen B., Schultz G., Harrison J., Hill R., Howell M., Speyrer M., Utra H., de Leon J., Lee W., Treadwell T. (2017). Oxidized regenerated cellulose/collagen dressings: review of evidence and recommendations. Adv. Skin Wound Care.

[65] Bode W., Meyer E., Powers J.C. (1989). Human leukocyte and porcine pancreatic elastase: x-ray crystal structures, mechanism, substrate specificity, and mechanism-based inhibitors. Biochemistry.

[66] Kim H.S., Sun X., Lee J.H., Kim H.W., Fu X., Leong K.W. (2019). Advanced drug delivery systems and artificial skin grafts for skin wound healing. Adv. Drug Deliv. Rev..

[67] Wolf M.T., Ganguly S., Wang T.L., Anderson C.W., Sadtler K., Narain R., Cherry C., Parrillo A.J., Park B.V., Wang G., Pan F., Sukumar S., Pardoll D.M., Elisseeff J.H. (2019). A biologic scaffold-associated type 2 immune microenvironment inhibits tumor formation and synergizes with checkpoint immunotherapy. Sci. Transl. Med..

[68] Cronce M.J., Faulknor R.A., Pomerantseva I., Liu X.H., Goldman S.M., Ekwueme E.C., Mwizerwa O., Neville C.M., Sundback C.A. (2018). In vivo response to decellularized mesothelium scaffolds. J. Biomed. Mater. Res. B Appl. Biomater..

[69] Delgado L.M., Fuller K., Zeugolis D.I. (2017). Collagen cross-linking: biophysical, biochemical, and biological response analysis. Tissue Eng. A.

[70] Ludolph I., Fried F.W., Kneppe K., Arkudas A., Schmitz M., Horch R.E. (2018). Negative pressure wound treatment with computer-controlled irrigation/instillation decreases bacterial load in contaminated wounds and facilitates wound closure. Int. Wound J..

[71] Pérez-Köhler B., Bayon Y., Bellón J.M. (2015). Mesh infection and hernia repair: a review. Surg. Infect..

[72] Fahrenbach E.N., Qi C., Ibrahim O., Kim J.Y., Alam M. (2013). Resistance of acellular dermal matrix materials to microbial penetration. JAMA Dermatol..

[73] Chen Y., Dan N., Dan W., Liu X., Cong L. (2019). A novel antibacterial acellular porcine dermal matrix cross-linked with oxidized chitosan oligosaccharide and modified by in situ synthesis of silver nanoparticles for wound healing applications. Mater. Sci. Eng. C.

[74] Zhou H.Y., Zhang J., Yan R.L., Wang Q., Fan L.Y., Zhang Q., Wang W.J., Hu Z.Q. (2011). Improving the antibacterial property of porcine small intestinal submucosa by nano-silver supplementation: a promising biological material to address the need for contaminated defect repair. Ann. Surg..

[75] Vercellotti G., McCarthy J., Lindholm P., Peterson P., Jacob H., Furcht L. (1985). Extracellular matrix proteins (fibronectin, laminin, and type IV collagen) bind and aggregate bacteria. Am. J. Pathol..

[76] Vytrasova J., Tylsova A., Brozkova I., Cervenka L., Pejchalova M., Havelka P. (2008). Antimicrobial effect of oxidized cellulose salts. J. Ind. Microbiol. Biotechnol..

[77] Steukers L., Glorieux S., Vandekerckhove A.P., Favoreel H.W., Nauwynck H.J. (2012). Diverse microbial interactions with the basement membrane barrier. Trends Microbiol..

[78] LeBleu V.S., MacDonald B., Kalluri R. (2007). Structure and function of basement membranes. Exp. Biol. Med..

[79] Olivero D., Furcht L. (1993). Type IV collagen, laminin, and fibronectin promote the adhesion and migration of rabbit lens epithelial cells in vitro. Invest. Ophthalmol. Vis. Sci..

[80] Woodley D., Wynn K., O'Keefe E. (1990). Type IV collagen and fibronectin enhance human keratinocyte thymidine incorporation and spreading in the absence of soluble growth factors. J. Invest. Dermatol..

[81] Kubo M., Kan M., Isemura M., Yamane I., Tagami H. (1987). Effects of extracellular matrices on human keratinocyte adhesion and growth and on its secretion and deposition of fibronectin in culture. J. Invest. Dermatol..

[82] Bissell D., Stamatoglou S., Nermut M., Hughes R. (1986). Interactions of rat hepatocytes with type IV collagen, fibronectin and laminin matrices. Distinct matrix-controlled modes of attachment and spreading. Eur. J. Cell Biol..

[83] Svensson A., Nicklasson E., Harrah T., Panilaitis B., Kaplan D.L., Brittberg M., Gatenholm P. (2005). Bacterial cellulose as a potential scaffold for tissue engineering of cartilage. Biomaterials.

[84] Huleihel L., Dziki J.L., Bartolacci J.G., Rausch T., Scarritt M.E., Cramer M.C., Vorobyov T., LoPresti S.T., Swineheart I.T., White L.J., Brown B.N., Badylak S.F. (2017). Macrophage phenotype in response to ECM bioscaffolds. Semin. Immunol..

[85] Sridharan R., Cavanagh B., Cameron A.R., Kelly D.J., O'Brien F.J. (2019). Material stiffness influences the polarization state, function and migration mode of macrophages. Acta Biomater..

[86] Rayahin J.E., Gemeinhart R.A., Kloc M. (2017). Activation of macrophages in response to biomaterials. Macrophages: Origin, Functions and Biointervention.

[87] Huard T., Malinoff H., Wicha M. (1986). Macrophages express a plasma membrane receptor for basement membrane laminin. Am. J. Pathol..

[88] Chen J., Cárcamo J., Bórquez-Ojeda O., Erdjument-Bromage H., Tempst P., Golde D. (2003). The laminin receptor modulates granulocyte-macrophage colony-stimulating factor receptor complex formation and modulates its signaling. Proc. Natl. Acad. Sci. U. S. A..

[89] Ohki K., Kohashi O. (1994). Laminin promotes proliferation of bone marrow-derived macrophages and macrophage cell lines. Cell Struct. Funct..

[90] Digiacomo G., Tusa I., Bacci M., Cipolleschi M., Dello Sbarba P., Rovida E. (2017). Fibronectin induces macrophage migration through a SFK-FAK/CSF-1R pathway. Cell Adhes. Migrat..

[91] Abdolghafoorian H., Farnia P., Sajadi Nia R.S., Bahrami A., Dorudinia A., Ghanavi J. (2016). Effect of Heart Valve Decellularization on Xenograft Rejection.

[92] Sgarioto M., Vigneron P., Patterson J., Malherbe F., Nagel M.-D., Egles C. (2012). Collagen type I together with fibronectin provide a better support for endothelialization. Comptes Rendus Biol..

[93] Underwood P.A., Bennett F.A. (1993). The effect of extracellular matrix molecules on the in vitro behavior of bovine endothelial cells. Exp. Cell Res..

[94] Kimura I., Yanagita S., Kobayshi S., Fukuta M., Okabe M. (2000). Vascular endothelial growth factor-and platelet-derived growth factor-angiogenesis depressed but fetal bovine serum-angiogenesis enhanced choroidal tissue cultures of. Int. Angiol..

[95] Castellon R., Hamdi H.K., Sacerio I., Aoki A.M., Cristina Kenney M., Ljubimov A.V. (2002). Effects of angiogenic growth factor combinations on retinal endothelial cells. Exp. Eye Res..

[96] Akpek E.K., Alkharashi M., Hwang F.S., Ng S.M., Lindsley K. (2014). Artificial corneas versus donor corneas for repeat corneal transplants. Cochrane Database Syst. Rev..

