# Supplementary material for: Porcine mesothelium matrix as a biomaterial for wound healing applications
Source: Mater Today Bio. 2020 May 17;7:100057. doi: 10.1016/j.mtbio.2020.100057 (PMC7305392; doi:10.1016/j.mtbio.2020.100057)
Supplement: Multimedia component 1 [file mmc1.docx]

**Supplementary Information**

Title

Porcine mesothelium matrix as a biomaterial for wound healing applications

Authors

Héctor Capella-Monsonís (1, 2), Maura A. Tilbury (2, 3), J. Gerard Wall (2, 3), Dimitrios I. Zeugolis (1, 2)^[[1]](#footnote-1)^

Affiliations

(1) Regenerative, Modular & Developmental Engineering Laboratory (REMODEL), National University of Ireland Galway (NUI Galway), Galway, Ireland

(2) Science Foundation Ireland (SFI) Centre for Research in Medical Devices (CÚRAM), National University of Ireland Galway (NUI Galway), Galway, Ireland

(3) Department of Microbiology, National University of Ireland Galway (NUI Galway), Galway, Ireland

**Supplementary Figure S1:** Immunocytochemistry analysis [cytoskeleton with rhodamine (red) and nuclei with Hoechst (blue)] made apparent a very low dermal fibroblast proliferation rate on CORC-PG. Scale bars 100 *µ*m. Overall quantification is provided at **Figure 6A**. SR: serosa side; PL: papillae side; CT: connective tissue side; BM: basement membrane side.


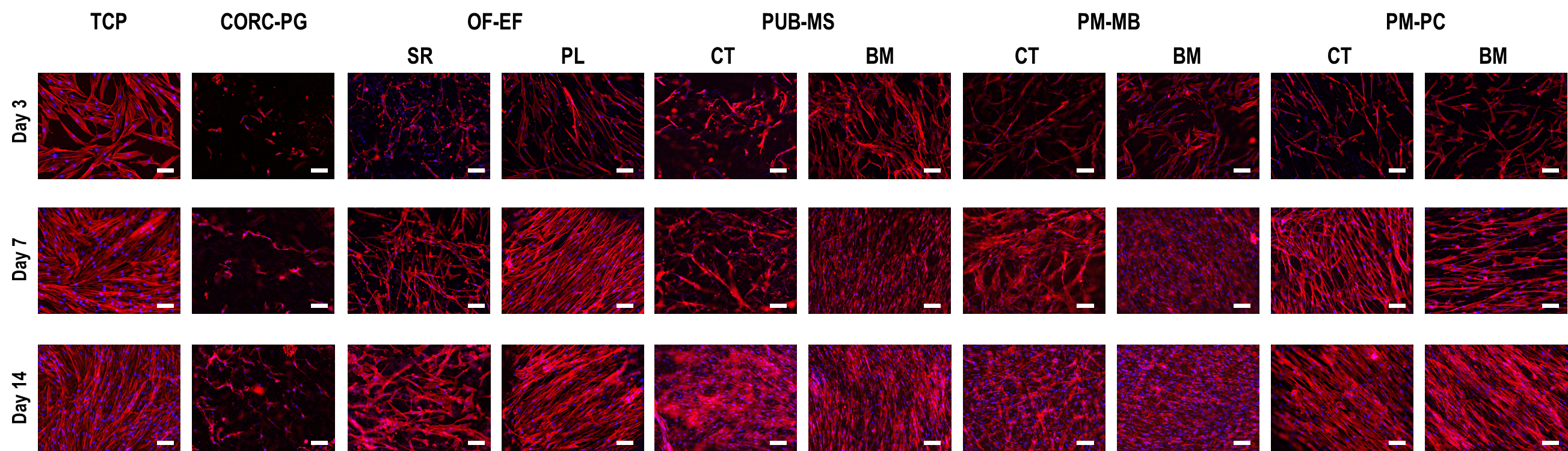


Supplementary Figure S2: Immunocytochemistry analysis for alive (calcein AM, green) and dead (ethidium homodimer, red) dermal fibroblasts on the various materials and time points. Scale bars 100 *µ*m. Overall quantification is provided at Figure 6C. SR: serosa side; PL: papillae side; CT: connective tissue side; BM: basement membrane side.


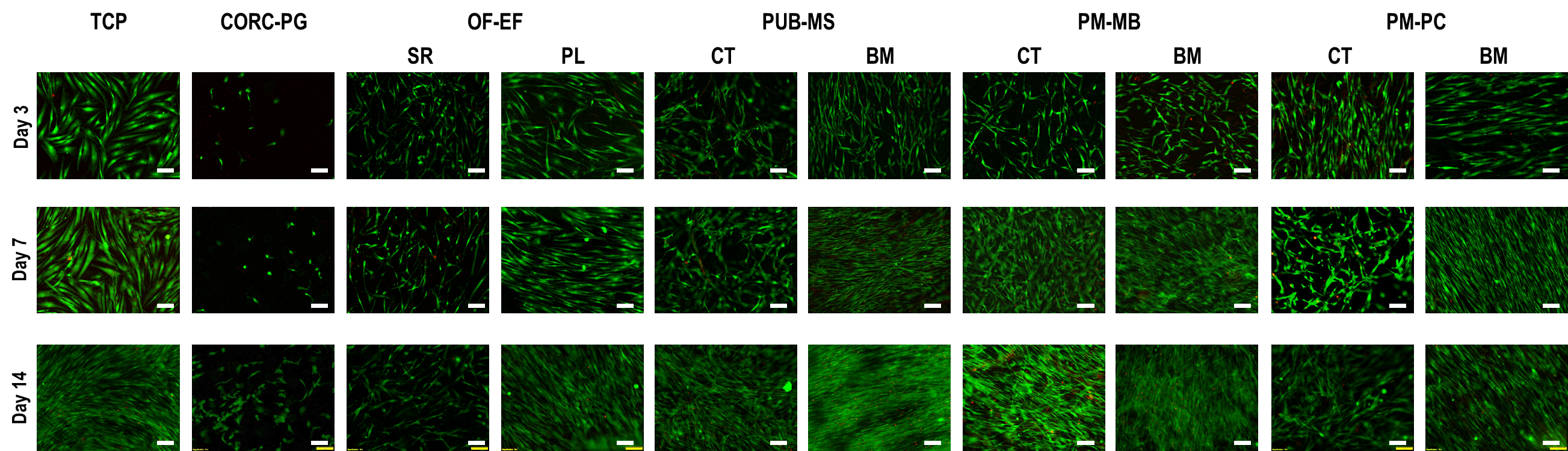


Supplementary Figure S3: Immunocytochemistry analysis for alive (calcein AM, green) and dead (ethidium homodimer, red) THP-1 monocytes on the various materials and time points. Scale bars 100 *µ*m. Overall quantification is provided at Figure 7C. SR: serosa side; PL: papillae side; CT: connective tissue side; BM: basement membrane side.


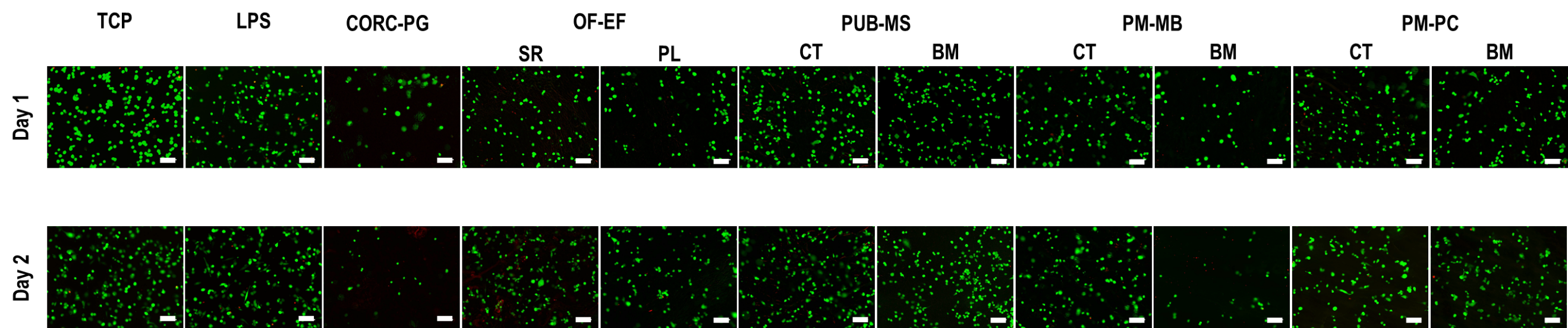


Supplementary Figure S4: Quantification of THP-1 proliferation, metabolic activity, cell viability and TNF-α production under materials conditioned media incubation for 2 days. This figure complements the results shown at Figure 7 and Figure 8. Data showed as average ± standard deviation (n=5).


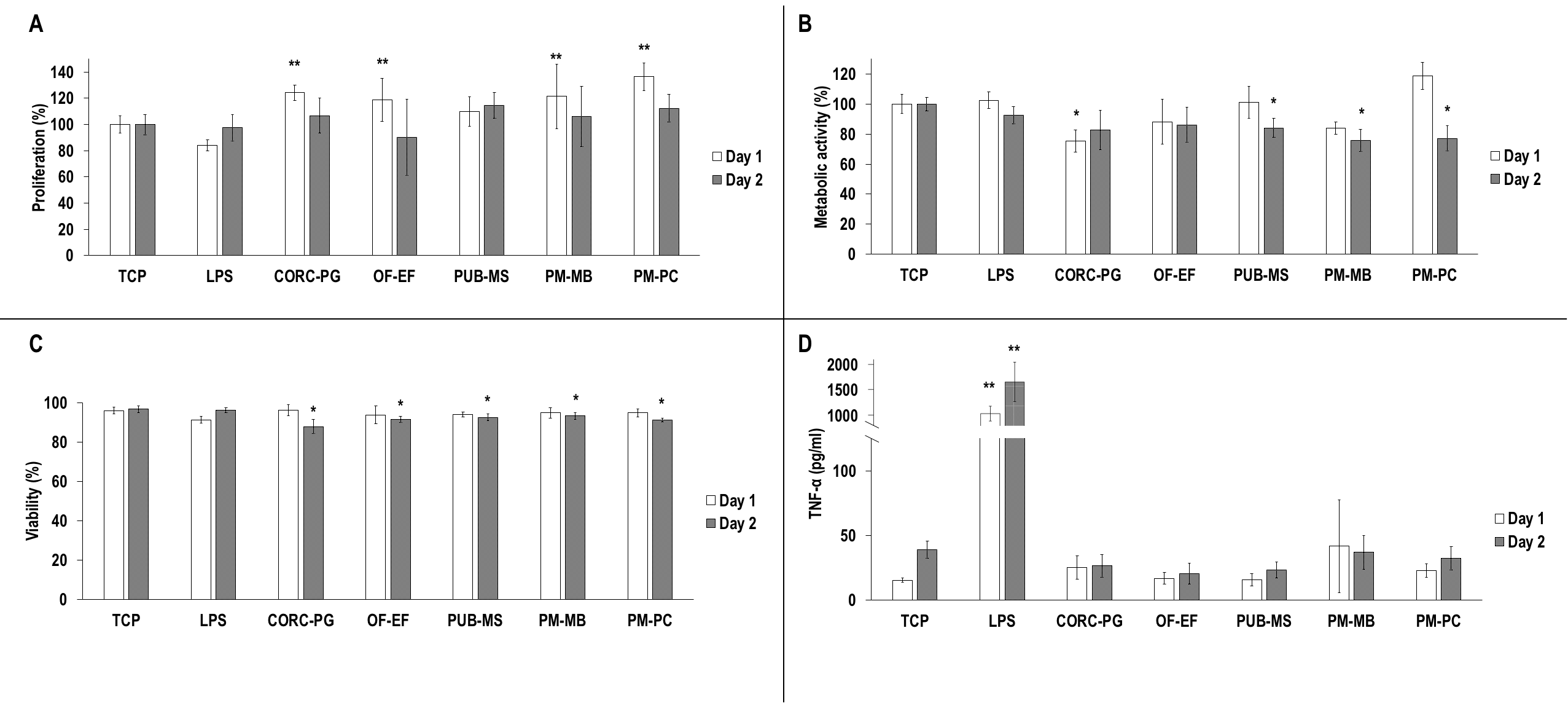


Supplementary Figure S5: Microscopy images of the scratch assay with HUVECs in media conditioned with different materials, where white dashed line indicates the front of cells. Overall quantification is provided at Figure 9A. Scale bars 100 *µ*m

1. Corresponding Author: Dimitrios I. Zeugolis, REMODEL, NUI Galway, Galway, Ireland. Telephone: +353 (0) 9149 3166; Email: dimitrios.zeugolis@nuigalway.ie [↑](#footnote-ref-1)
